# Alternate Modes of Photosynthate Transport in the Alternating Generations of *Physcomitrella patens*

**DOI:** 10.3389/fpls.2017.01956

**Published:** 2017-11-13

**Authors:** Kamesh C. Regmi, Lin Li, Roberto A. Gaxiola

**Affiliations:** School of Life Sciences, Arizona State University, Tempe, AZ, United States

**Keywords:** *Physcomitrella patens*, symplasmic transport, apoplasmic transport, transfer cells, sucrose synthase pathway, sucrose/H^+^ symporter, P-type H^+^ ATPase, proton pyrophosphatase

## Abstract

*Physcomitrella patens* has emerged as a model moss system to investigate the evolution of various plant characters in early land plant lineages. Yet, there is merely a disparate body of ultrastructural and physiological evidence from other mosses to draw inferences about the modes of photosynthate transport in the alternating generations of *Physcomitrella*. We performed a series of ultrastructural, fluorescent tracing, physiological, and immunohistochemical experiments to elucidate a coherent model of photosynthate transport in this moss. Our ultrastructural observations revealed that *Physcomitrella* is an endohydric moss with water-conducting and putative food-conducting cells in the gametophytic stem and leaves. Movement of fluorescent tracer 5(6)-carboxyfluorescein diacetate revealed that the mode of transport in the gametophytic generation is symplasmic and is mediated by plasmodesmata, while there is a diffusion barrier composed of transfer cells that separates the photoautotrophic gametophyte from the nutritionally dependent heterotrophic sporophyte. We posited that, analogous to what is found in apoplasmically phloem loading higher plants, the primary photosynthate sucrose, is actively imported into the transfer cells by sucrose/H^+^ symporters (SUTs) that are, in turn, powered by P-type ATPases, and that the transfer cells harbor an ATP-conserving Sucrose Synthase (SUS) pathway. Supporting our hypothesis was the finding that a protonophore (2,4-dinitrophenol) and a SUT-specific inhibitor (diethyl pyrocarbonate) reduced the uptake of radiolabeled sucrose into the sporangia. *In situ* immunolocalization of P-type ATPase, Sucrose Synthase, and Proton Pyrophosphatase – all key components of the SUS pathway – showed that these proteins were prominently localized in the transfer cells, providing further evidence consistent with our argument.

## Introduction

The advent of colonization and the subsequent diversification of the early ancestral lineages of Embryophyta (Kenrick and Crane, 1997; Qiu et al., 2006) elicited a period of unprecedented morphological innovations among early land plants. Phylum Bryophyta forms the basal land plant lineage (Aoki et al., 2003), possesses simple morphology, and represents the ancestral land plant archetype (Renzaglia et al., 2000). Given that these contemporary bryophytes represent distant descendants of early land plants from which modern tracheophytes were also derived (Hébant, 1977), these plants offer a unique prospect to examine the early morphological adaptations required for existence on land (Renzaglia et al., 2000). Of these morphological innovations, the evolution of vascular tissues is considered one of the most important events in plant evolutionary history (Prigge and Bezanilla, 2010).

Among the species constituting mosses, *Physcomitrella patens* belongs to Funariaceae, and was the first bryophyte to have its complete genome sequenced (Rensing et al., 2008). This is particularly relevant because, as a non-vascular land plant, *Physcomitrella* lies at the evolutionary interface between aquatic green algae and vascular plants, and as such, is well-suited to allow comparisons that can yield phylogenetic insights into the genomic changes associated with the occupation of land (Rensing et al., 2008). Yet, the physiological aspects of photosynthate transport and partitioning in *Physcomitrella* have not been reported – with only a disparate body of ultrastructural and physiological evidence from other mosses to extrapolate from.

Like all mosses, *Physcomitrella* gametophore lacks true vascular tissue. The gametophore stem, however, possesses a relatively complex stem anatomy with a central water-conducting strand composed of xylem analogs called hydroids (Sakakibara et al., 2003). Whether the stem of *Physcomitrella* gametophore also has food-conducting cells is as yet unknown. Nevertheless, endohydric mosses like *Physcomitrella* often also possess specialized food-conducting cells, and these cells have been interpreted to resemble phloem sieve cells (Behnke, 1975; Behnke and Sjolund, 2012). These food-conducting cells, also referred to as leptoids (Hébant, 1970, 1975; Behnke, 1975), are considered analogs of sieve elements (Ligrone et al., 2000), and have been postulated to form a symplasmic conduit for the movement of sucrose in the gametophore (Reinhart and Thomas, 1981; Raven, 2003). Intriguingly, the *Physcomitrella* genome harbors orthologs of H^+^-coupled sucrose uptake transporters (SUTs) (Kühn and Grof, 2010; Reinders et al., 2012a), and given that the mode of photosynthate transport in mosses is likely to be symplasmic (Raven, 2003), it has been argued that the SUTs in moss gametophores in fact act to recover sucrose leaked into the apoplasm (Reinders et al., 2014). In addition to SUTs, six orthologous sequences for Sugars Will Eventually be Exported Transporter (SWEET) proteins are also found in the *Physcomitrella* genome (Eom et al., 2015), which alludes to the existence of an apoplasmic route of sucrose transport.

The symplasmic mode of photosynthate transport, however, only accounts for the dominant haploid gametophytic generation of mosses and ignores the transient diploid sporophytic generation. This is especially important because it is widely maintained that the largely heterotrophic sporophyte is nutritionally dependent on the photosynthetic gametophyte (Ligrone and Gambardella, 1988; Ligrone et al., 1993). This functional dependence is clearly mirrored in the morphological specializations of the transfer cells that lie at the interface between the sporophyte and the gametophyte (Ligrone and Gambardella, 1988; Ligrone et al., 1993). These transfer cells are characterized by their extensive cell wall invaginations, dense ribosome-rich cytoplasm, and abundant mitochondria (Pate and Gunning, 1972; Browning and Gunning, 1979a; Lal and Chauhan, 1981; Ligrone et al., 1993). Furthermore, in *Physcomitrella*, the sporophyte-gametophyte junction presents a diffusion barrier, i.e., the metabolites have to cross an apoplasmic boundary (Courtice et al., 1978). This implies that the acropetal translocation of sucrose across the gametophyte-sporophyte interface (i.e., the haustorium) is likely to involve an apoplasmic loading mechanism, with the metabolic energy for this directional movement of sucrose being supplied by the numerous transfer cell mitochondria. Having said this, however, the literature is ambivalent on the mechanism of sucrose movement from the gametophyte to the sporophyte in mosses.

The transfer cells in mosses are characterized by intense enzymatic activity (Hebant and Suire, 1974; Chauhan and Lal, 1982), and corollarily, Browning and Gunning (1979b,c) showed using excised haustoria of *Funaria hygrometrica* that both externally applied glucose and sucrose influx into the haustoria was inhibited by metabolic uncouplers. Conversely, Renault et al. (1992) showed that there is a so-called non-continuity of sucrose at the gametophyte-sporophyte junction, with hexoses being the predominant sugars at this interface in *Polytrichum formosum* (Renault et al., 1992). In fact, Renault et al. (1989, 1992) claimed that the proton motive force established at the plasma membrane of transfer cells in the haustorium does not play a major role in sucrose uptake, but rather in amino acid uptake (Renault et al., 1989, 1992). Their physiological studies instead pointed to a mechanism in which cell wall invertases hydrolyze sucrose into hexoses, with these hexoses being reconverted to sucrose upon absorption into the haustorium (Renault et al., 1992). In a preceding study, the same authors showed that, at maturity, the apoplasmic boundary between the sporophyte and gametophyte in *Polytrichum* is acidic with a pH of ∼4.30 (Renault et al., 1989).

Under the scenario proposed by Renault et al. (1989, 1992), the mechanism of sucrose transport from the gametophyte to the sporophyte would be independent of the activity of SUS in the transfer cells (Renault et al., 1989, 1992). However, a repertoire of enzyme assays in the gametophytic and sporophytic tissues of several mosses revealed substantial activities of all the enzymes associated with the SUS pathway (Galloway and Black, 1989). Additionally, given that the invertase pathway of sucrose metabolism is twice as costly in terms of ATP consumption compared to the pyrophosphate (PPi)-dependent SUS pathway (Black et al., 1987), it is plausible that mosses use the more energy-efficient mode of SUS metabolism to nourish the sporophytic tissue. This, in turn, would entail that PPi homeostasis is crucial for the proper functioning of moss transfer cells, analogous to what is observed in the phloem of apoplasmically phloem loading species *Arabidopsis thaliana* (Pizzio et al., 2015).

Given the incongruent body of ultrastructural and physiological evidence from other mosses, the genomic evidence from *Physcomitrella*, and the ancient evolutionary origin of proteins like SUTs that are directly implicated in apoplasmic phloem loading in vascular plants, we deemed it plausible to use the non-vascular model moss *Physcomitrella* to directly examine the route and the key components involved in photosynthate transport in both the gametophytic and sporophytic generations. Specifically, we wanted to address questions like: Were proteins like SUTs, P-type ATPases, H^+^-PPase, and SUS, neo-functionalized in the vascular tissues of higher plants, or are there evolutionarily conserved roles of such proteins that transcend the phylogenetic diversity of land plants? Does an apoplasmic mechanism of sucrose transport predate the evolution of tracheophytes?

To elucidate a cogent model of photosynthate transport in the alternating generations of *Physcomitrella*, we gathered evidence from ultrastructural, physiological, fluorescent tracing, and immunohistochemical studies. Our investigations suggest that there are alternate modes of photosynthate transport in the alternating generations of *Physcomitrella*, wherein sucrose transport is predominantly passive and symplasmic in the gametophytes, while sucrose is actively loaded apoplasmically into the transfer cells via the SUS pathway in the sporophytic generation.

## Materials and Methods

### Culture Conditions

The Gransden wild-type strain from *Physcomitrella patens* Bruch and Schimp was used for this study. Gametophytes were either grown on solid BCD media in petri plates, or on soil fertilized by liquid BCD media, in a growth chamber with 16 h light and 8 h dark regime (Cove et al., 2009). Sporophyte growth was induced by growing 4–5 weeks old mature gametophytes in a chamber held at 15°C under 8 h of light and 16 h of darkness.

### Light Microscopy

Individual mature *Physcomitrella* gametophytes were imaged using an Olympus SZX7 stereomicroscope (Olympus Corporation, Tokyo, Japan) equipped with an Infinity 2 camera system and Infinity Capture imaging software (Lumenera Corporation, Ottawa, Canada).

Resin-embedded naked stems were prepared for transmission electron microscopy (described below) and cut into semi-thin (250–500 nm) sections, stained with toluidine blue, and imaged using a Nikon Eclipse TE300 inverted microscope (Nikon Corporation, Tokyo, Japan) equipped with an Olympus DP26 camera and cellSens digital imaging software (Olympus Corporation, Tokyo, Japan).

### Transmission Electron Microscopy

Individual mature *Physcomitrella* gametophores were fixed in 4.0% glutaraldehyde and 1.0% formaldehyde in 0.05 M sodium phosphate buffer, pH 7.2 for 2 h at room temperature. The leaves were gently separated from several stems under a stereomicroscope while still in the fixative fluid, and both leafy and naked stems were dissected into ∼2 mm pieces. The leaves and stems were further fixed in fresh, buffered fixative for 2 h at room temperature, post-fixed with 1% osmium tetroxide (OsO_4_) for 2 h at room temperature, and *en bloc* stained with 0.2% uranyl acetate overnight at 4°C. The tissues were then serially dehydrated in acetone, gradually infiltrated with Spurr’s resin over 5–7 days, and flat-embedded in molds or on Teflon-coated glass slides in a 60°C oven. The tissues were embedded in various orientations to facilitate longitudinal and transverse sectioning. The resin blocks were trimmed, cut into 70–100 nm sections with a diamond knife (Diatome Inc., Hatfield, PA, United States) in a Leica Ultracut R microtome (Leica Microsystems, Vienna, Austria), mounted onto formvar-coated copper slot or 100-mesh grids (Electron Microscopy Sciences, Washington, PA, United States), and post-stained with 2% methanolic uranyl acetate and Sato’s lead citrate. The sections were then imaged at 80 kV using JEOL 1200EX transmission electron microscope (JEOL Ltd., Tokyo, Japan) equipped with SIA model L3C bottom-mount air-cooled CCD camera (Scientific Instruments and Applications, Inc., Duluth, GA, United States) and Maxim DL5 imaging software (Diffraction Ltd., Ottawa, ON, Canada). All the fixative chemicals were purchased from Electron Microscopy Sciences (Washington, PA, United States).

### Scanning Electron Microscopy

Individual gametophores and sporophytes were fixed with 4% glutaraldehyde in 0.05 M sodium phosphate buffer, pH 7.2 for 4–6 h at room temperature, post-fixed in 1% OsO_4_ for 2 h at room temperature, serially dehydrated with acetone, and critical point dried (Balzers Union, Principe de Liechtenstein). If the tissues were to be dissected, the critically dried samples were cut in a desirable fashion using a fresh double-edged razor blade under a stereomicroscope. The samples were then mounted onto aluminum stubs, gold-palladium sputter-coated (Technics, Alexandria, Virginia), and imaged at 15 kV using JEOL JSM-6300 scanning electron microscope (JEOL Ltd., Tokyo, Japan) equipped with IXRF digital acquisition system (IXRF Systems, Inc., Austin, TX, United States). All the fixative chemicals were purchased from Electron Microscopy Sciences (Washington, PA, United States).

The scanning and transmission electron micrographs (SEMs and TEMs, respectively) were then processed using Adobe Photoshop CC software (Adobe Systems Incorporated, San Jose, CA, United States). Linear adjustments (brightness, contrast, and levels) were made to the entire images to enhance clarity.

### Fluorescent Tracer Study

Individual gametophores or sporophyte-bearing gametophores were vertically floated on ∼5 μL of 250 μg.mL^-1^ 5(6) carboxyfluorescein diacetate (CFDA) or 250 μg.mL^-1^ HPTS solution inside PCR tube strips, and incubated for 1, 3, or 24 h in a dark humid chamber. The plants were then rinsed in excess volume of double-distilled water at least three times, and directly whole-mounted on slides with water as a mounting medium, and imaged with an AMG EVOS FL Cell Imaging epifluorescent system (Life Technologies Inc., United States) using in-built 4X/0.13 NA objective lens, GFP excitation (470 nm) and emission (525 nm) light cube, and brightfield optics. Negative controls were performed by floating the plants in water only. The sporophyte-bearing gametophores were identically processed, except that most of the leaves were excised prior to imaging. For CFDA tracer study in individually excised leaves, single leaves were carefully removed from the stem using fine forceps under a stereomicroscope, and processed identically as above except only 1 – 2 μL of CFDA solution was used.

### Aniline Blue Staining and Confocal Microscopy

Aniline blue staining of plasmodesmata-associated callose of *Physcomitrella* leaves were performed according to the protocol described by Zavaliev and Epel (2015), using 0.01% (w/v) aniline blue in 0.01 M K_3_PO_4_, pH 12. The leaves were then whole-mounted in the staining solution itself and imaged with a Leica TCS SP5 AOBS Spectral Confocal microscope (Leica Microsystems) equipped with a 20×/0.7 NA Plan-Apo dry objective lens, DIC optics, and Leica confocal software. Plasmodesmata-associated aniline blue staining was imaged (512 pixels × 512 pixels) at an excitation wavelength of 405 nm, and the emission signal was detected between 475 and 525 nm for aniline blue, and between 643 and 730 nm for chlorophyll autofluorescence. Negative controls were performed by excluding aniline blue.

### Immunohistochemistry

Entire gametophores and sporangia-bearing gametophores were immediately immersed in at least 20-fold excess volume of FAA (10% v/v 37% formaldehyde : 5% v/v acetic acid: 50% v/v 200-proof ethanol : 35% v/v water) fixative, and placed under a gentle vacuum for 10 – 15 min at room temperature. The fixation was then allowed to continue overnight at 4°C. Following this, the tissues were dehydrated in a graded ethanol series, exchanged with xylene to clear the tissues, and gradually infiltrated with paraplast. The tissues were then embedded in paraplast in desired orientations. Using a Leica RM2155 rotary microtome (Leica Microsystems, Germany),^1^ the tissue blocks were cut into 10 μm thick sections and mounted on poly-L-Lysine coated slides. The tissues were then deparaffinized, rehydrated, and exposed to an antigen retrieval buffer (Biogenex, United States)^2^ in a 65°C water bath for 20 min. After washing in water, the endogenous peroxidase activity was quenched using 3% (v/v) H_2_O_2_. The tissues were washed with Phosphate Buffered Saline, pH 7.2 with 0.01% (v/v) Tween-20 (PBST), and blocked with 1% (w/v) casein in PBST for 60 min at room temperature. After washing with PBST three times, the tissues were exposed to anti-AVP1, anti-SUS, or anti-AHA3 rabbit polyclonal antibodies at 1:1000 dilutions overnight at 4°C. Negative controls were concurrently performed using pre-immune serum at 1:1000 dilutions in PBST, or by excluding the primary antibody. After two washes with PBST and one wash with PBS, the signal was developed using the SignalStain^®^ Boost IHC HRP anti-rabbit detection system (Cell Signaling Technology, United States),^3^ following the manufacturer’s instructions. To develop the signal when mouse anti-serum was used, the DAKO EnVISION anti-rabbit anti-mouse system was used according to the manufacturer’s instructions (DAKO Inc.).^4^ The tissues were then run through a dehydrating ethanol series, and were permanently mounted. Images were acquired with a Zeiss Axioskop light microscope (Carl Zeiss Inc.) equipped with 10× Achroplan 0.25 NA Zeiss, 40× Plan Neo-Fluar 0.75 NA Zeiss, and 100× 1.30 NA Oil Plan Neo-Fluar objective lenses, phase and differential interference contrast optics, Olympus DP72 camera system, and Olympus cellSens^®^ imaging software. Alternatively, a Nikon Eclipse E600 microscope equipped with Nikon Plan Fluor 10× 0.30 NA, 20× 0.50 NA, 100× 1.30 NA Oil objective lenses (Nikon Instruments Inc.), Omax A355OU camera (OMAX Microscopes), and ToupView software (ToupTek Photonics) was used.

### Total Protein Extraction

Approximately 300 mg of *Physcomitrella* gametophores were immediately ground into fine powder with liquid N_2_ on a cold mortar and pestle. The liquid N_2_ was allowed to evaporate, and the fresh frozen powder was transferred in ∼200 μL aliquots to screw-cap eppendorf tubes. To each tube, 1 mL of 10% (v/v) Trichloroacetic acid in -20°C acetone was added, and proteins were allowed to precipitate overnight at -20°C. The samples were centrifuged at 10,000 × *g* for 30 min at 4°C, followed by removal of the supernatant. The pellet was washed with -20°C acetone containing 0.07% β-mercaptoethanol, vortexed, and centrifuged at 10,000 × *g* for 10 min at 4°C. The washing, vortexing, and centrifugation steps were repeated four more times. After the final centrifugation step, the supernatant was removed and the pellet dried in a tabletop vacuum for ∼30 min. Total protein was solubilized by adding Laemmli’s buffer to the pellet. Prior to storage at -80°C the mixture was vortexed and centrifuged at 5,000 × *g* for 5 min at 4°C three times.

### Western Blot

The solubilized protein extracts from were then run on a 10% SDS-polyacrylamide gel, transferred to a PVDF membrane, blocked with 5% (w/v) non-fat milk in Tris buffered saline with 0.01% Tween-20 (TBST), and probed with H^+^-PPase-specific polyclonal sera or anti-AHA3 polyclonal sera at 1:1000 dilution overnight at 4°C. After three washes with TBST, the membrane was developed using the Bio-Rad Alkaline Phosphatase Immun-Blot^®^ Colorimetric Assay kit (Bio-Rad Inc., United States)^5^ according to the manufacturer’s instructions.

### Soluble Carbohydrate Measurement

Approximately 100 mg of gametophores were immediately ground to fine powder using liquid N_2_ in pre-chilled mortar and pestle. The liquid N_2_ was allowed to evaporate, and the fresh frozen powder aliquoted into five pre-chilled screw-cap eppendorf tubes. Then 250 μL of 80% ethanol was added to each tube, vortexed, incubated at 80°C for 20 min with shaking (100 rpm) in Eppendorf Thermomixer R (Eppendorf Co.), and centrifuged at 14,000 rpm for 5 min at 4°C. The supernatants from the tubes were transferred to fresh pre-cooled eppendorf tubes, and these tubes were kept on ice. To the pellet, 150 μL 80% ethanol was added, vortexed, incubated at 80°C for 20 min with shaking (100 rpm) in Eppendorf Thermomixer R, followed by centrifugation at 14,000 rpm for 5 min at 4°C. The supernatants from the tubes were then pooled with the respective supernatants from the first step. Finally, 250 μL of 50% ethanol was added to the pellet, vortexed, incubated at 80°C for 20 min in the Eppendorf Thermomixer R, and centrifuged at 14,000 rpm for 5 min at 4°C. Again, the supernatants were pooled into respective tubes. The soluble carbohydrate content, i.e., sucrose, glucose, and fructose, was spectrophotometrically determined using the Megazyme^®^ Sucrose/D-Fructose/D-Glucose Assay Kit (Megazyme Inc.) following the manufacturer’s instructions.

### Radioisotope Labeling with ^14^CO_2_

To determine the uptake of photosynthate by the heterotrophic sporangia, ^14^CO_2_ labeling was performed on sporophyte-bearing gametophytes. *Physcomitrella patens* were grown on 10 of the 18 wells in an 18-well plate under 16 h/8 h light/dark period at 22°C until the gametophores attained maturity (∼5–6 weeks) and then transferred to 8 h/16 h light/dark period at 15°C until the gametophores bore sporangia. Approximately 20 sporangia-bearing gametophores were used for each time point. The experiment was performed when the sporangia were still green. The 18-well plate containing the plants was kept under a halogen lamp (∼500 lumens) for 2 h to acclimatize the plants to the lighting conditions, and then kept in a sealed transparent box prior to radioisotope labeling. A micro-blower (Pelonis Technologies Inc.) powered by an external battery pack was affixed inside the box to homogeneously circulate the air, and later ^14^CO_2_. ^14^CO_2_ was injected into the box by mixing 2.5 μL of NaH^14^CO_3_ (specific activity: 56 mCi/mmol, concentration: 2.0 mCi/mL; MP Biomedicals) with an excess of 80% lactic acid in a syringe. Photosynthesis was allowed to take place for 20 min, after which the ^14^CO_2_ was vented, and the box kept in the dark. At specific time points (1, 3, 5, 9, and 25 h) two colonies were taken out of the wells, and only the sporangia were excised under a stereomicroscope and immediately transferred to two scintillation vials (∼15 sporangia per vial) containing 100 μL of 80% methanol to stop metabolism. Then 50 μL of commercial bleach was added to the vials, and finally 5 mL of scintillation cocktail (MP Biomedicals) was added to each of the vial. After the mixture had cleared, scintillation counting was performed in a Beckman Coulter LS6500 liquid scintillation counter.

### [U^14^C]-Sucrose and [U^14^C]-Maltose Uptake and Inhibitor Study

Given that both CFDA and HPTS moved freely through the gametophore, with their movement only halted at the gametophore-sporophyte interface, we argued that exogenously supplied radiolabeled sugars would also move similarly. [U^14^C]-sucrose (specific activity: 432 mCi.mmol^-1^; concentration: 100 μCi.mL^-1^; MP Biomedicals) and [U^14^C]-maltose (specific activity: 600 mCi.mmol^-1^; concentration: 100 μCi.mL^-1^; American Radiolabeled Chemicals Inc.) were prepared at final concentrations of 0.1 μCi.mL^-1^ in a solution containing 0.5 mM CaCl_2_, 0.25 mM MgCl_2_, and 1 mM “cold” sucrose. 2,4-DNP was prepared in the same solution, with or without [U^14^C]-sucrose at final concentrations of 5, 1, and 0.5 mM. DEPC was prepared identically at final concentrations of 1.7 and 0.5 mM. For all the assays, individual sporophyte-bearing gametophores were floated on a 5 μL drop of experimental solution in PCR tube strips inside a humid chamber, and allowed to take up the assay solution for 4 h in the dark. For [U^14^C]-sucrose and [U^14^C]-maltose assays, the mosses were individually pretreated on 5 μL drops of solution containing 0.5 mM CaCl_2_ and 0.25 mM MgCl_2_ for 1 h before transferring them to solutions containing radiolabeled sugars. Similarly, for the inhibitor treatments, the mosses were incubated on 5 μL drops of solution containing 0.5 mM CaCl_2_ and 0.25 mM MgCl_2_ with aforementioned inhibitor concentrations for 1 h before transferring them to solutions with both labeled and unlabeled sucrose and the specific inhibitor. After 4-h long incubation in the dark, individual sporangium was excised under a stereomicroscope and immediately transferred to a scintillation vial containing 100 μL of 80% methanol. Then 50 μL of commercial bleach was added to the vials, and finally, 5 mL of scintillation cocktail (MP Biomedicals) was added to each of the vials. After the mixture had cleared, scintillation counting was performed in a Beckman Coulter LS6500 liquid scintillation counter.

### Immunogold Labeling and Transmission Electron Microscopy

High-pressure freezing (HPF) and freeze-substitution (FS): Haustoria from gametophytes with fully grown sporophytes were excised under a drop of 1-hexadecene, and quickly loaded into interlocking brass planchettes, filled with 1-hexadecene, and subjected to HPF in a BalTec HPM 010 high-pressure freezer. The cryofixed haustoria were then transferred to a pre-chilled -80°C FS cocktail containing anhydrous acetone and 0.2% (w/v) non-methanolic uranyl acetate. The FS was allowed to continue for 96 h at -80°C, after which the FS solution was allowed to warm to 4°C over a period of 12 h. The anhydrous acetone was exchanged with anhydrous ethanol several times, and the tissues were gradually infiltrated with medium grade London Resin (LR) White (Electron Microscopy Sciences, United States) at 4°C: 25% (v/v) LR White (12 h), 50% (v/v) LR White (12 h), 75% (v/v) resin (12 h), 100% LR White (12 h), 100% LR White (12 h), 100% LR White (12 h). The samples were transferred to gelatin capsules, filled with fresh LR White and polymerized for 24 h at 50°C in longitudinal orientation. The polymerized resin blocks were then trimmed, ultrathin sectioned to gold reflectance (90 – 100 nm) using a Leica Ultracut R ultramicrotome (Leica Microsystems, Germany), and mounted on formvar-coated nickel slot grids.

For immunogold labeling, the nickel slot grids containing the sections were floated on drops of blocking solution (1% w/v casein in PBST) for 1 h at room temperature. The grids were then sequentially transferred to drops of PBST before being floated on drops of anti-AVP1 or anti-SUS polyclonal antibodies diluted 10-fold in the blocking solution. The antibody incubation step was performed at 4°C overnight. Following this, the grids were washed five times with PBST, and then transferred to drops of anti-rabbit IgG conjugated to either Aurion Ultrasmall 0.8 nm (H^+^-PPase) or 10 nm (SUS) gold particles (Electron Microscopy Sciences, United States) diluted 25- to 50-fold in the blocking solution. The grids were further washed in PBST five times, and rinsed with double-distilled water three times, before being air-dried overnight. Silver enhancement of Aurion Ultrasmall immunogold labeled sections was performed for 3 min in a dark room by using HQ SILVER^TM^ Enhancement kit (Nanoprobes Inc.) following the manufacturer’s instructions. The sections were then imaged with a JEOL 1200EX transmission electron microscope (JEOL Corp., Japan) operating at 80 kV without post-staining. Linear image adjustments like contrast, brightness, and levels were adjusted on entire grayscale electron micrographs using Adobe Photoshop CC (Adobe Inc., United States).

### Molecular Phylogenetic Analysis

The amino acid sequences for H^+^PPase and SUS orthologs in *P. patens* were identified by BlastP in www.cosmoss.org or www.gramene.org, using the reference sequences from *A. thaliana*,^6^ *Vigna radiata*,^7^ and *Oryza sativa*.^8^ The amino acid sequences were first aligned using MUSCLE built-into the MEGA5 software, and then the evolutionary history was inferred by using the Maximum Likelihood method based on the JTT matrix-based model (Jones et al., 1992). The bootstrap consensus tree was deduced from 500 replicates. Evolutionary analyses were conducted in MEGA5 (Tamura et al., 2011).

## Results

### Morphology of *Physcomitrella* Gametophyte and Sporophyte

**FIGURE 1:**
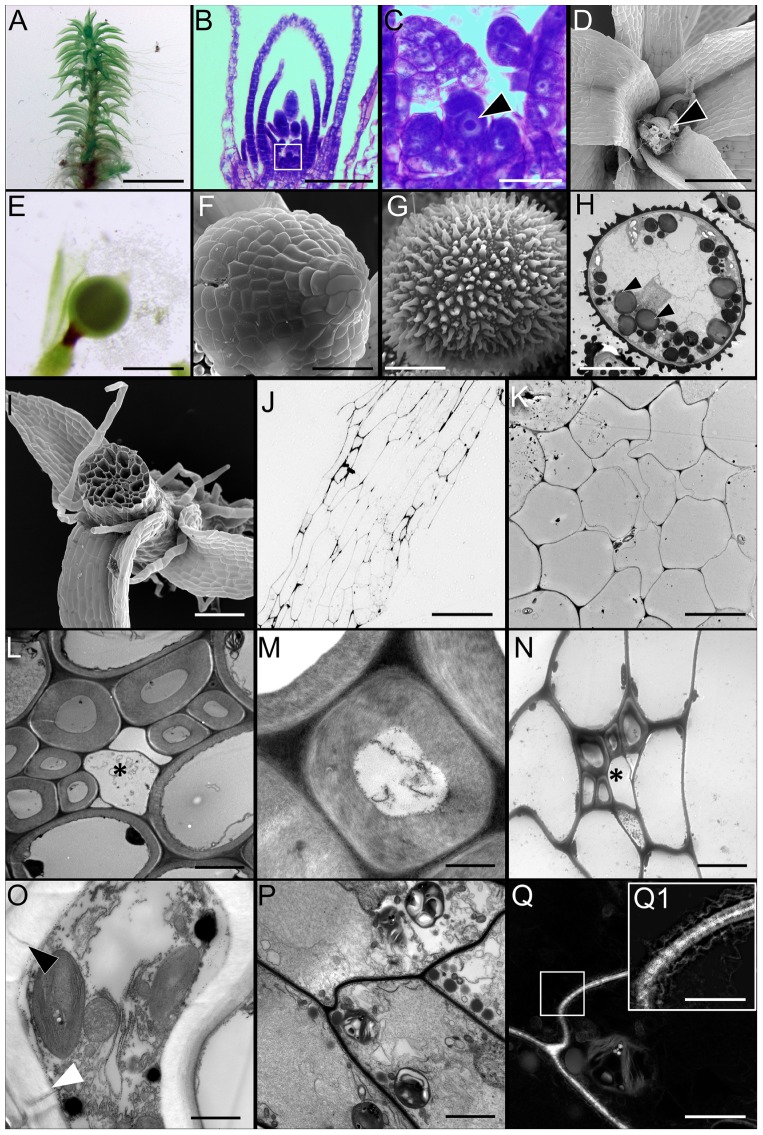
Morphology of *Physcomitrella patens* and the ultrastructure of conducting cells. **(A)** Light micrograph (LM) of a representative mature leafy gametophore used for this study. **(B,C)** LM of apical region of the gametophore stained with toluidine blue. Note the single apical meristematic cell in (**C**, arrowhead; inset from **B**). **(D)** Scanning electron micrograph (SEM) of the apical region of a mature gametophyte. Notice the female archegonia (arrowhead). **(E)** LM of sporophyte with an apical immature sporangium. **(F)** SEM of a sporangium. **(G)** SEM of a typical haploid spheroidal spore, with prominent exine ornamentation. **(H)** Transmission electron micrograph (TEM) of a spore showing the internal ultrastructure. Numerous osmiophilic lipid storage globules are present (arrowheads). **(I)** SEM of gametophore stem in cross-section showing the overall organization of the conducting cells. The distinctive central bundle of hydroids is clearly visible. **(J)** LM of the near median longitudinal section through the stem showing the general organization of the elongated conducting cells. The centrally located cells are hydroids, while the directly adjoining cells are food-conducting cells. **(K)** TEM of cross-section of stem showing the centrally located thin-walled hydroids devoid of cytoplasmic contents. **(L)** TEM of transverse section through leaf costa showing the adaxially located thick-walled stereid cells, with three empty thin-walled hydroids in the center (^∗^). **(M)** Higher magnification TEM of a thick-walled stereid cell. The narrow lumen is mostly empty, and the cell wall has a loose, fibrillar structure. **(N)** TEM of transverse section through the stem showing a leaf trace right beneath the cortical epidermal layer, with thick-walled stereids, and thin-walled hydroids (^∗^). **(O)** TEM of a putative food-conducting cell in the leaf midrib with plasmodesmata in the lateral wall (arrowheads). **(P)** Longitudinal TEM section through the stem depicting putative food-conducting cells. **(Q)** Inverted grayscale image of **(P)**, with the boxed inset **(Q1)** showing a higher magnification image of the end wall profusely decorated with plasmodesmata. A higher magnification grayscale image of **(P)** was inverted in Adobe Photoshop CC to generate **(Q)**. The 2048 pixel × 2048 pixel image in **(Q)** was digitally zoomed and cropped (inset, **Q**), and the Levels and Unsharp Mask functions applied to reveal the plasmodesmata and the associated endoplasmic reticular network seen in **(Q1)**. Scale bars: **(A)** 5 mm, **(B)** 50 μm, **(C)** 10 μm, **(D)** 100 μm, **(E)** 1 mm, **(F)** 200 μm, **(G)** 25 μm, **(H)** 25 μm, **(I)** 300 μm, **(J)** 200 μm, **(K)** 5 μm, **(L)** 5 μm, **(M)** 1 μm, **(N)** 5 μm, **(O)** 5 μm, **(P)** 10 μm, **(Q)** 15 μm, **(Q1)** 10 μm.

Although mosses undergo an alternation of generations like all land plants, they differ from vascular plants in that the haploid gametophytic stage, rather than the diploid sporophytic stage, is the dominant phase of the life cycle. In *Physcomitrella*, the gametophores (**Figure 1A**) emerge from protonemal filaments and are affixed to the substrate by basal filamentous rhizoids. There is a single apical meristematic cell that gives rise to young phyllids (**Figures 1B,C**), as the gametophore undergoes vertical growth. At the apex of the monoecious gametophore, archegonia containing the female gamete or egg (**Figure 1D**) and antheridia containing male gametes, or sperm form. Upon fertilization, an apical sporophyte (**Figures 1E,F**) is formed within each archegonium. The diploid tissue in this structure, the capsule undergoes meiosis to form numerous haploid spheroidal spores, with a characteristic exine layer (**Figures 1G,H**).

The *Physcomitrella* stem is an anatomically complex structure, and possesses a well-developed central column of water-conducting cells or hydroids (**Figures 1I–K**), corroborating a previous report (Sakakibara et al., 2003). These cells are thin-walled, and are devoid of living protoplasm at maturity, presumably due to programmed cytoplasmic lysis (**Figure 1K**) (Hebant and Johnson, 1976). The midrib or costa of leaves, in cross-section, reveals that the central region of the midrib is mostly comprised of thick-walled parenchyma or stereid cells (**Figure 1L**). These stereid cells have narrow lumens, and do not retain living protoplasts at maturity (**Figure 1M**). Given the thickness of their cell walls, it seems plausible that the function of the stereid cells is limited to the mechanical support of the leaf. The thick walls of stereid cells show loose, fibrillar fine structure, and their function in internal conduction of water cannot be ruled out (**Figure 1M**). In the leaf costa, thin-walled hydroids lie abaxial to the stereids, and are mostly empty (**Figure 1L**). The leaves penetrate the stem in the cortical layer, just below the epidermis, forming a leaf trace (**Figure 1N**). The presence of small, thick-walled stereid cells, and thin-walled empty hydroids characterize the leaf trace (**Figure 1N**). These cells are presumably contiguous with their respective counterparts in the leaf midrib. The cross-section of *Physcomitrella* leaf costa shows a putative food-conducting cell, with plasmodesmatal connections to the laterally adjoining cells (**Figure 1O**). The stems of *Physcomitrella* have elongate putative food-conducting cells with oblique end walls that are profusely decorated with plasmodesmata (**Figures 1P–Q1**). The presence of plasmodesmata suggests that the mechanism of sucrose translocation via these food-conducting cells is symplasmic in nature (Raven, 2003).

### Symplasmic Translocation in the Gametophytic Stem and Leaves of *Physcomitrella*

**FIGURE 2:**
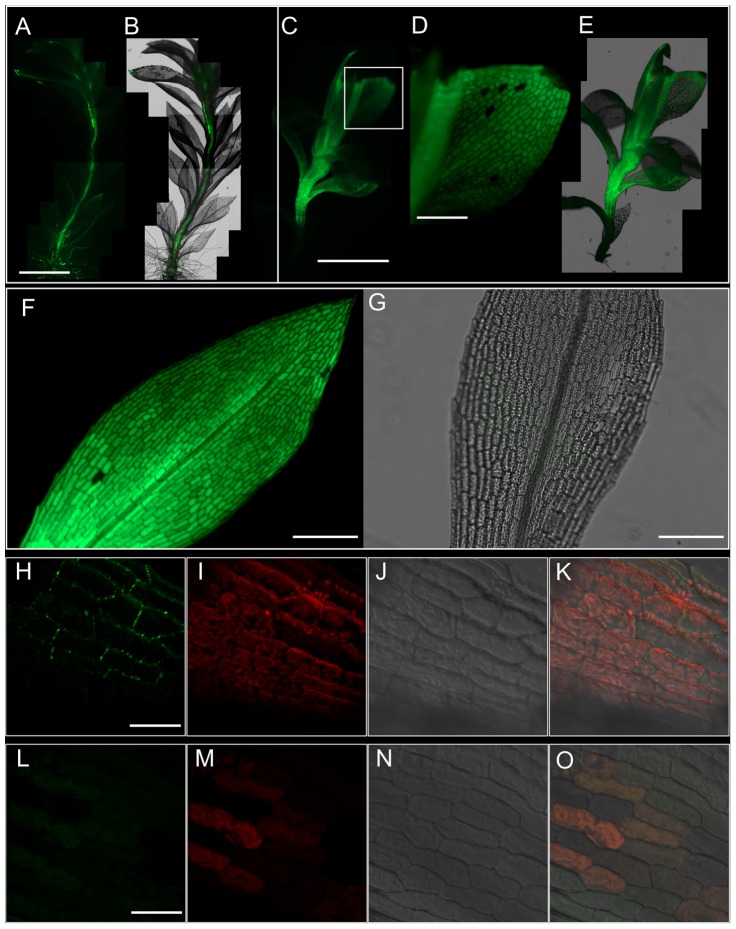
Symplasmic translocation in the *Physcomitrella* gametophyte stem and leaves. **(A)** Montage of epifluorescence micrographs showing the translocation of 5(6)-carboxyfluorescein diacetate (CFDA) to the apex of the gametophore after 1 h of CFDA application from the rhizoidal end of the stem. Excitation wavelength (Ex): 470 nm, Emission wavelength (Em): 525 nm. **(B)** Overlay of **(A)** on the brightfield micrograph of the gametophore. **(C)** Whole-mount montage of epifluorescent micrographs of a gametophore 24 h after treatment with CFDA from the rhizoidal end. The fluorescence has spread throughout the stem. **(D)** Higher magnification image from (C; inset) showing the uniform distribution of CFDA in the leaf. **(E)** Superimposition of **(C)** on the brightfield micrograph of the gametophyte. **(F)** Focus stacked composite of 12 epifluorescence micrographs of an excised leaf fed with CFDA from the base for 12 h, showing the homogeneous distribution of CFDA throughout the leaf. Focus stacking was performed in Adobe Photoshop CC. **(G)** An overlay of images from the brightfield and GFP channels of a representative negative control showing the lack of any interfering autofluorescence. **(H–K)** Maximum projection confocal micrographs of leaf laminar cells stained with alkalinized aniline blue to visualize plasmodesmata-associated callose. A distinct punctate staining pattern diagnostic of plasmodesmata can be clearly seen (green; **H**). Autofluorescence from chloroplasts is also visible (red; **I**), while **(J)** shows the differential interference contrast (DIC) image of the same image field. **(K)** An overlay of images from **(H–J)**. Aniline blue Ex: 405 nm; Em: 475–525 nm. Chloroplast Ex: 405 nm; Em: 643–730 nm. **(L–O)** Negative control for aniline blue staining, showing no callose staining **(L,O)**, and imaged under the same conditions. Scale bars: **(A,B)** 2.5 mm, **(C,E)** 5 mm, **(D)** 500 μm, **(F,G)** 500 μm, **(H–K)** 10 μm, **(L–O)** 10 μm.

Using a fluorescent tracer 5(6)-carboxyfluorescein diacetate (CFDA), we studied whether the translocation of photosynthate in the gametophytic generation of *Physcomitrella* was symplasmic in nature. After 1 h of application from the rhizoidal end, CFDA was found to have translocated to the tip of the leafy gametophores (**Figures 2A,B**), providing support for our ultrastructural observation (**Figure 1Q**). Upon observing the movement of CFDA in the gametophores 24 h after application, the fluorescence was spread throughout the stem (**Figures 2C,E**), and the leaves (**Figure 2D**). These results directly suggested that a symplasmic continuum exists in the *Physcomitrella* gametophyte.

We further analyzed the movement of CFDA in individually excised leaves, and 12 h after application of CFDA from the bottom of the leaves, the fluorescence was uniformly spread through all the leaf cells including the midrib (**Figure 2F**), while a negative control did not show any interfering autofluorescence (**Figure 2G**). This finding suggested plasmodesmatal interconnections among leaf cells, and upon callose-specific aniline blue staining, a distinct punctate fluorescent pattern was observed (**Figures 2H–K**). Considered a diagnostic test for the presence of plasmodesmata, aniline blue staining in the leaves of *Physcomitrella* gave further proof that the leaf cells of *Physcomitrella* are symplasmically connected. As expected, a negative control experiment showed no plasmodesmata-specific aniline blue staining (**Figures 2L–O**).

### *Physcomitrella* Sporophyte Is Symplasmically Isolated from the Gametophyte

**FIGURE 3:**
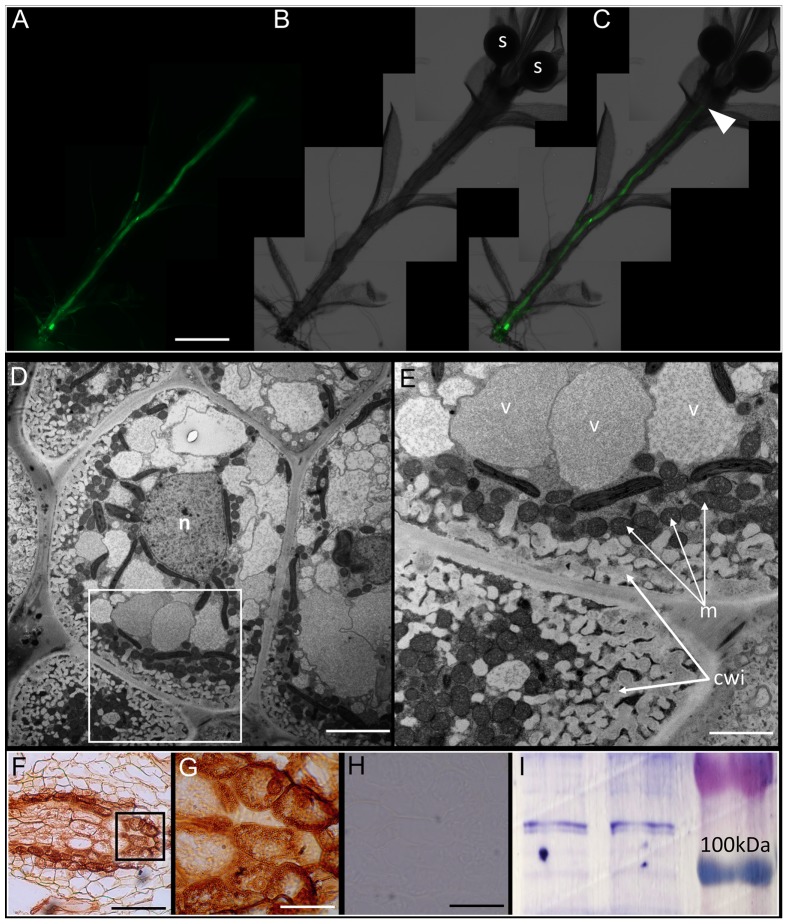
*Physcomitrella* sporophyte is symplasmically isolated from the gametophyte by transfer cells. **(A)** Montage of whole-mount epifluorescence micrographs of a sporophyte-bearing gametophore showing the movement of 5(6)-carboxyfluorescein diacetate (CFDA) to the gametophyte-sporophyte interface 3 h after CFDA application from the rhizoidal end. **(B)** Brightfield micrograph of the gametophore showing the sporangia (labeled s), **(C)** Overlay of **(A)** and **(B)** showing the approximate location of haustorium (arrowhead) where CFDA movement stopped. **(D)** Ultrastructure of the haustorium – the diffusion barrier identified in **(A–C)** – showing the presence of transfer cells, with a large central nucleus (n). **(E)** Higher magnification image of the inset in **(D)** showing the characteristic cell wall invaginations (cwi) and proximally located mitochondria (m). **(F)** Immunohistochemical localization of P-type ATPase orthologs of companion cell-specific *Arabidopsis* H^+^-ATPase 3 (AHA3) in transfer cells that constitute the haustorium. The localization is identified by the distinctive brown 3,3′-diaminobenzidine precipitate formed upon catalysis by secondary antibody conjugated horse radish peroxidase (see Materials and Methods). **(G)** Inset from **(F)** showing a higher magnification image of the transfer cells with P-type ATPase localization. **(H)** Representative negative control with primary antibody excluded, showing a lack of signal. **(I)** Cross-reactivity of anti-AHA3 antisera with total protein extracts from *Physcomitrella* showing identifiable bands >100 kDA that correspond to P-type ATPase orthologs with predicted sizes of 106 kDa. Scale bars: **(A–C)** 1 mm, **(D)** 5 μm, **(E)** 1 μm, **(F)** 100 μm, **(G)** 20 μm, **(H)** 50 μm.

A previous study reported that a diffusion barrier exists between the gametophytic and sporophytic generation of *Physcomitrella* (Courtice et al., 1978). We fed the sporophyte-bearing gametophores with CFDA from the rhizoidal end, and 3 h after application, it was found that the movement of CFDA was halted at an interface separating the sporophyte from the gametophore (**Figures 3A–C**). Identical results were obtained when HPTS was used as a fluorescent tracer (Supplementary Figures S1A–C). To further verify that the movement of CFDA was symplasmic in the sporophyte-bearing *Physcomitrella* gametophore stem, we pretreated the plants with 2,4-DNP, a metabolic uncoupler, and found that CFDA was translocated unimpeded through the gametophore stem (Supplementary Figures S1F,G), and that a diffusion barrier existed between the haploid and diploid generations of *Physcomitrella*.

In bryophytes, the sporophyte-gametophyte junction called the haustorium, has long been known to harbor a band of transfer cells (Ligrone and Gambardella, 1988; Ligrone et al., 1993). The name of the cells itself implies their presumptive role in the ‘transfer’ of metabolites and nutrients. Light microscopic evidence for the existence of transfer cells at this interface in *Physcomitrella* exists (Xu et al., 2014), but electron microscopic evidence has been lacking. We analyzed the haustorium using a conventional chemical fixation regime, and the distinctive cytological features of transfer cells were evident (**Figures 3D,E**). Saliently, the transfer cells had intricate cell wall invaginations with numerous mitochondria in their immediate vicinity – morphological attributes seen as directly facilitating the energy-intensive nature of nutrient transfer via an increase in the absorptive surface area and proximally located sources of primary energy currency ATP (**Figure 3E**). A large central nucleus, several vacuoles, and plastids were also observed (**Figures 3D,E**).

Given the proximity of numerous mitochondria to the cell wall invaginations (**Figure 3E**), we posited that P-type ATPases must be localized in the haustorium. Using polyclonal rabbit antibodies generated against the KLH-conjugated TISKDRVKPSPTPDS epitope of *A. thaliana* companion cell-specific AHA3 protein (DeWitt et al., 1991; DeWitt and Sussman, 1995; Pizzio et al., 2015), we tested the cross-reactivity of the antibody against total protein extracts from *Physcomitrella* by western blotting, and found a distinct band (>100 kDa) corresponding to P-type ATPase orthologs in the moss (**Figure 3I**). *In silico* analysis revealed 8 putative orthologs of ATPases in *Physcomitrella*, and that the antigenic epitope was indeed highly conserved (67–93%) in this moss (Supplementary Figure S3A). And as expected, P-type ATPases were prominently immunolocalized at the haustorium, suggesting a proton gradient energized uptake of nutrients via the transfer cells (**Figures 3F,G**). The specificity of the primary antibody was confirmed by excluding the primary antibody, which yielded no staining (**Figure 3H**).

### Apoplasmic Loading of Sucrose from the Gametophyte to the Sporophyte

**FIGURE 4:**
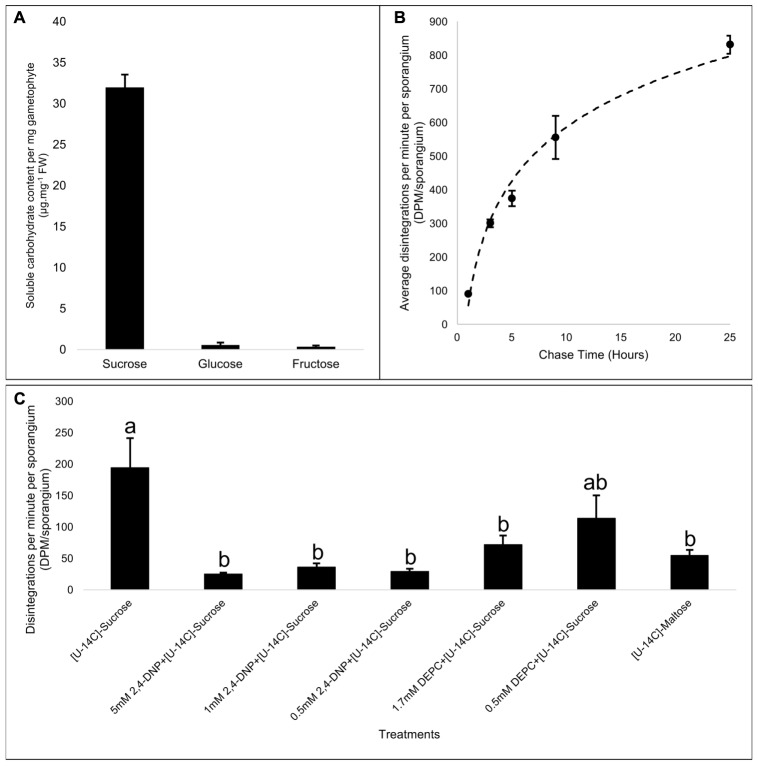
Apoplasmic loading of the primary photosynthate, sucrose, into the *Physcomitrella* sporophyte. **(A)** Spectrophotometric quantitation of net content of principle soluble sugars – sucrose, glucose, and fructose – per mg fresh weight mass of gametophore, shows that sucrose is by far the predominant sugar, and is likely the primary photosynthate being transported. **(B)** Average radioactive label detected in sporangia of mosses labeled for 20 min in the light by ^14^CO_2_, and chased for specific time points in the dark (1, 3, 5, 9, and 25 h), showing a saturable kinetics of photosynthate assimilation into the heterotrophic sporangia. *n* = 2 pools per time point; ∼15 sporangia per pool; variation represented by standard error bars. **(C)** Effect of a protonophore, 2,4-dinitrophenol (2,4-DNP), and sucrose/H^+^ symporter (SUT)-specific inhibitor diethyl pyrocarbonate (DEPC) on the transport of exogenously supplied [U^14^C]-sucrose into the sporangia. Compared to control (i.e., [U^14^C]-sucrose alone), 2,4-DNP effectively inhibited sucrose movement into the sporangia at all concentrations tested (5, 1, and 0.5 mM). DEPC was much more effective in inhibiting sucrose transport into the sporangia at a concentration of 1.7 mM than at 0.5 mM. [U^14^C]-maltose, a substrate for SUTs, is also transported to the sporangia, but only at levels nearly three times less than the control. *F*_6,28_ = 6.75, *p* < 0.0002, variation represented by standard error bars. It was inferred from *post hoc* pairwise comparisons using Tukey HSD that the mean disintegrations per minute per sporangium (DPM.sp^-1^) for the control (mean = 194.4, *SE* = 46.8) was significantly different from the DPM.sp^-1^ of [U^14^C]-sucrose + 5 mM 2,4 DNP (mean = 24.9, *SE* = 2.4), [U^14^C]-sucrose + 1 mM 2,4 DNP (mean = 36.2, *SE* = 5.8), [U^14^C]-sucrose + 0.5 mM 2,4 DNP (mean = 29.5, *SE* = 4.0), [U^14^C]-sucrose + 1.7 mM DEPC (mean = 72.0, *SE* = 14.3), and [U^14^C]-maltose (mean = 54.88, *SE* = 8.6), while other pairwise comparisons did not yield statistically significant differences at the 95% confidence interval. Different letters over bars, a or b, indicate significant differences.

To test for the physiological aspects of photosynthate movement between the two generations of *Physcomitrella*, we first analyzed the soluble carbohydrate content in the gametophore, and in agreement with previous reports (Allsopp, 1951; Thelander et al., 2009), found that sucrose was by far the predominant soluble carbohydrate (**Figure 4A**). Given the likely nutritional dependency of the sporophytic generation on the photoautotrophic gametophyte (Ligrone and Gambardella, 1988; Ligrone et al., 1993), we postulated that the growing sporangium of *Physcomitrella* was a strong heterotrophic sink tissue. While immature sporangia are green in color (**Figure 1E**), and likely photosynthetic, the surface area to volume ratio imparted by its spherical structure is not ideal for maximal photosynthetic capacity. We labeled immature sporophyte-bearing *Physcomitrella* gametophores with ^14^CO_2_ for 20 min, and chased the radioactive pulse sequestered in the sporangia at several time points (1, 3, 5, 9, and 25 h) over a period of 25 h in the dark (**Figure 4B**). Within the 1st hour, a minimal amount of label was measured in the sporangia, suggesting that these structures were indeed minimally photosynthetic. Over the course of 25 h, it was found that more and more radioactive label had moved into the sporangia, and that the kinetics of the photosynthate uptake into the sporangia was likely saturable (**Figure 4B**).

The set of evidence obtained thus far painted a picture wherein the sporangia were heterotrophic sinks, and that the primary photosynthate sucrose was apoplasmically loaded via the transfer cells. This implied that, like in apoplasmically loading higher plant species like *A. thaliana*, the uptake of sucrose into the transfer cells was mediated by sucrose/proton symporters (SUTs) powered by the proton gradient generated by P-type ATPases. Therefore, we used a protonophore 2,4-DNP, and a SUT-specific inhibitor DEPC (Bush, 1993) to investigate their effects on the uptake of exogenously applied [U-^14^C]-sucrose into the growing sporangia. 2,4-DNP was effective in significantly diminishing sucrose uptake into the sporangium at all the concentrations (5, 1, and 0.5 mM) tested (**Figure 4C**). It has been known that DEPC specifically modifies the histidine residue located in the first extracellular loop near the second transmembrane domain of the SUT, and is accessible from the extracellular face of the plasma membrane (Bush, 1993; Lu and Bush, 1998), and upon bioinformatics analysis, it was found that of the six SUT paralogs harbored in the *Physcomitrella* genome, this histidine residue was fully conserved among all of them (Supplementary Figure S3C). Accordingly, application of 1.7 mM DEPC significantly decreased the exogenous sucrose uptake into the sporangium, while 0.5 mM DEPC, while reducing the mean sucrose uptake, was not significantly effective (**Figure 4C**).

Phylogenetic analysis has shown that *Physcomitrella* harbors both types II and III SUTs (Kühn and Grof, 2010; Reinders et al., 2012a), wherein the former subtype is localized to the plasma membrane and is generally implicated in phloem loading in monocots, while the latter are localized at the vacuoles and mediate sucrose uptake from the cytoplasm. Furthermore, given that the type II SUTs are also known to have a high affinity for maltose (Sivitz et al., 2005; Reinders et al., 2006, 2012b; Sun et al., 2009), we measured the uptake of [U-^14^C]-maltose into the sporangia, and found that the sporangia had taken up approximately threefold less maltose associated label than they did sucrose (**Figure 4C**). These results further strengthened our argument that the mechanism of sucrose movement into the sporangium via the transfer cells is empowered by ATPase generated proton gradient and is mediated by SUTs.

### Key Components of the Sucrose Synthase Pathway Immunolocalize at the Transfer Cells

**FIGURE 5:**
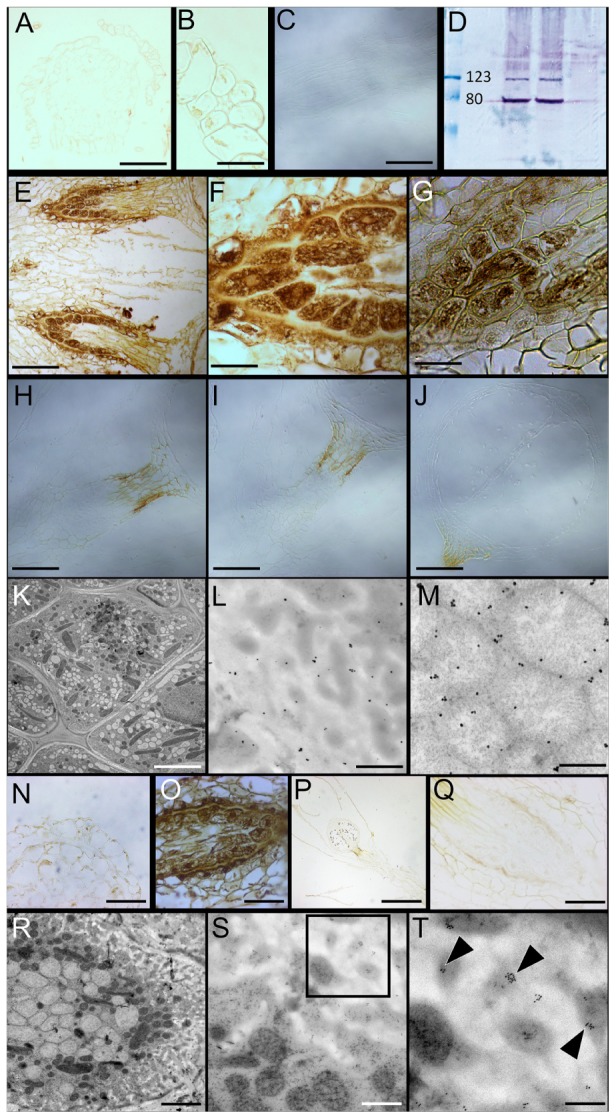
Immunolocalization of Proton Pyrophosphatase (H^+^-PPase) and SUS orthologs in *Physcomitrella* gametophytes and haustoria. **(A,B)** H^+^-PPases impart a poor immunohistochemical signal in the gametophore stem **(A)**, and leaves **(A,B)**. **(C)** Pre-immune sera showing no label in the gametophore stem. **(D)** Western blot of *Physcomitrella* total protein extracts interrogated with anti-H^+^-PPase sera showing the predicted distinctive band at ∼80 kDa. **(E,F)** H^+^-PPases are conspicuously localized at the gametophyte-sporophyte junction – the haustorium, characterized by transfer cells. **(G)** Immunolocalization of H^+^-PPases at the haustorium independently confirmed using antisera generated in mouse. **(H)** Pre-immune sera control, **(I)** Secondary antibody control, and **(J)** no labeling control. The brown coloration seen in **(H–J)** is an endogenous color of the seta as seen in **Figure 1E**. **(K–M)** Immunogold labeling followed by silver enhancement shows both plasma membrane **(L)** and vacuolar **(M)** localization of H^+^-PPases in high-pressure frozen/freeze substituted (HPF/FS) transfer cells **(K)**. **(N)** Like H^+^-PPases, SUS also imparts a very weak immunohistochemical signal in the gametophore stem and leaf. **(O)** Prominent SUS localization at the transfer cells lining the haustorium. **(P,Q)** Negative controls excluding the primary antibody showed no signal. **(R–T)** Immunogold labeling of SUS in the HPF/FS processed transfer cells **(R)** showing SUS-specific labeling in close proximity to the plasma membrane along the cell wall invaginations (**S** inset; **T** arrowheads). Scale bars: scale bars: **(A)** 100 μm, **(B)** 25 μm, **(C)** 100 μm, **(E)** 100 μm, **(F,G)** 20 μm, **(H–J)** 50 μm, **(K)** 5 μm, **(L,M)** 250 nm, **(N)** 25 μm, **(O)** 50 μm, **(P)** 200 μm, **(Q)** 50 μm, **(R)** 5 μm, **(S)** 1 μm, **(T)** 250 nm.

In apoplasmically phloem loading higher plant species, the sieve element-companion cell complexes are symplasmically isolated in the vascular bundles, and part of the actively symported sucrose is metabolized via the SUS pathway to supply ATP for the maintenance of proton gradient that, in turn, is used for sucrose symport via SUTs. There is a substantial body of evidence that implicates two enzymes – SUS and plasma membrane localized H^+^-PPase – as key components of this pathway (Lerchl et al., 1995; Paez-Valencia et al., 2011; Gaxiola et al., 2012, 2015; Pizzio et al., 2015; Regmi et al., 2015). As such, we immunolocalized these two enzymes in the haustorium/transfer cells at both the light and electron microscopy level (**Figure 5**).

H^+^-PPases are evolutionarily conserved proteins with a pyrophosphate-binding KAADVGADLVGKVE motif that is highly conserved among diverse organisms, including *P. patens* (Supplementary Figure S3B) (Rea and Poole, 1985; Rea et al., 1992). The antibody used in this study was raised against this motif in a rabbit (Park et al., 2005), and recognized the H^+^-PPase specific band (∼80 kDa) when immunoblotted against the total protein extract from *Physomitrella* (**Figure 5D**). In the sporophytic generation, marked by the development of the sporangium, a distinct immunohistochemical signal was observed at the sporophyte-gametophyte interface (**Figures 5E–G**). Specifically, localization of H^+^-PPases was evident at the transfer cells that characterize this filial junction (**Figure 5G**). Representative pre-immune and secondary antibody controls lacked any label (**Figures 5H–J**). Interestingly, the gametophore stem (**Figure 5A**) and leaves (**Figures 5A,B**) had relatively very weak signals. Pre-immune sera treated control, as expected, lacked any discernible signal (**Figure 5C**). A parallel immunohistochemical localization of H^+^-PPases using antisera raised against the KAADVGADLVGKVE motif in mouse (Paez-Valencia et al., 2011) was also performed, that further confirmed the transfer cell-specific localization of H^+^-PPases (**Figure 5G**).

Given the cytology of these transfer cells, and because these cells translocate sucrose (an endogenous cryoprotectant), and the need for immunogold labeling of these cells, we cryofixed the excised haustorium samples using HPF and freeze substitution (HPF/FS) regime. To determine the membrane-specificity of H^+^-PPases in transfer cells, silver-enhanced immunogold labeling was performed on high-pressure frozen samples (**Figure 5K**), and both the plasma membrane and the vacuoles were profusely decorated with H^+^-PPase-specific label (**Figures 5L,M**). Negative controls performed with pre-immune sera showed the specificity of the H^+^-PPase label (Supplementary Figures S2A–C). In parallel, immunohistochemical localization of SUS showed that it was prominently localized at the haustorium (**Figure 5O**), while the antibody-specific signal was very weak in the gametophyte stem and leaf (**Figure 5N**). The cross-reactivity of the antibody used against protein extracts from *Physcomitrella* was reported previously (Regmi et al., 2015). Negative controls that omitted the primary antibody showed no signal (**Figures 5P,Q**). Upon immunogold labeling of HPF/FS processed haustorium (**Figure 5R**) using the anti-SUS antibody showed clear labeling at the plasma membrane (**Figures 5S,T**), while the negative controls showed a clear lack of gold labeling (Supplementary Figures S2D,E).

## Discussion

As in all plants, sucrose (Suc) is the primary photosynthate in mosses (Allsopp, 1951). Likewise, the predominant soluble sugar in *Physcomtirella* is Suc (**Figure 4A**), congruent with its ability to use Suc as its carbon and energy source when grown in the absence of light but in the presence of exogenous Suc (Thelander et al., 2009). The *Physcomitrella* genome must, therefore, harbor all the genes required for Suc metabolism. In fact, two Suc biosynthetic enzymes, sucrose phosphate synthase (SPS) and sucrose-phosphate phosphatase (SPP), and Suc-degrading enzyme SUS are indeed encoded in the *Physcomitrella* nuclear genome (Thelander et al., 2009).

The entry of carbon from Suc into cellular metabolism in plants can be catalyzed by either SUS or invertase (INV) enzymes, wherein the former catalyzes the reversible conversion of Suc to fructose and UDP-glucose, while the latter catalyzes the irreversible hydrolysis of Suc to glucose and fructose (Huber and Akazawa, 1986; Black et al., 1987). The route of Suc catabolism is an important determinant in energy conservation and carbon allocation in non-photosynthetic cells, because the conversion of Suc to hexose phosphates via the SUS pathway uses half of the ATP that is required upon conversion via INV (Koch, 2004; Barratt et al., 2009).

*In silico* analysis has revealed that *Physcomitrella* genome contains eight putative neutral/alkaline INVs and six acid INVs (Thelander et al., 2009). Classified according to the pH optima of their enzymatic activities, these INVs also have different sub-cellular localization, with neutral/alkaline INVs generally thought to be located in the cytoplasm, while acidic INVs localize either to the cell wall apoplasm, or in the vacuoles (Roitsch and González, 2004). Interestingly, phylogenetic comparison of the acidic INV family in *Physcomitrella* with that of *Arabidopsis* revealed that all six of the *Physcomitrella* INVs group closer to their vacuolar orthologs in *Arabidopsis* (Thelander et al., 2009). Whether, by inference, this means that *Physcomitrella* does not have cell wall INVs needs further experimental validation.

Our evidence supports the idea that photosynthetic *Physcomitrella* gametophores primarily allocate and transport carbon in the form of Suc (**Figure 4**). In tracheophytes, the major forms of Suc transport involve energy-intensive apoplasmic, passive symplasmic, or polymer trapping processes (Turgeon, 1996; Turgeon and Ayre, 2005; Rennie and Turgeon, 2009). *Physcomitrella* gametophores, like many other mosses, only retain xylem and phloem analogs in the form of hydroids and food-conducting cells (**Figures 1I–Q**) (Ligrone et al., 2000). The profusion of plasmodesmatal pores in the end walls of the food-conducting cells (**Figure 1Q**), and the passive movement of the fluorescent tracers 5(6)-carboxyfluorescein diacetate (CFDA) and HPTS through the leaves and stem (**Figures 2**, **3** and Supplementary Figure S1) is consistent with the notion that Suc transport in the haploid *Physcomitrella* gametophores is predominantly symplasmic in nature (Raven, 2003).

However, the *Physcomitrella* genome also contains six putative Suc/H^+^ symporters (SUTs) (Reinders et al., 2014) – a finding that at first glance seems to be at odds with the symplasmic mode of Suc transport in the haploid gametophores. But this point of view ignores the transient diploid sporophytic generation of *Physcomitrella*, which our data (**Figure 4B**) and the published literature suggest to be a strong heterotrophic sink tissue (Ligrone and Gambardella, 1988; Ligrone et al., 1993). Furthermore, given that the photosynthetic gametophyte of *Physcomitrella* is symplasmically isolated from the sporophyte sink (**Figures 3A–E** and Supplementary Figure S1) (Courtice et al., 1978), it seems plausible that this intergenerational interface is the site for apoplasmic transfer of Suc from the gametophyte to the sporophyte. Adding credence to this notion was our finding that this filial junction in *Physcomitrella* has a band of transfer cells, and that P-type ATPase orthologs of companion cell-specific AHA3 protein are distinctly localized in these cells (**Figures 3D–G**).

Transfer cells are found in all plant taxonomic groups, as well as algae and fungi (Pate and Gunning, 1972; Offler et al., 2003), indicating that the genetic tools requisite for transfer cell morphogenesis evolved primitively (Pate and Gunning, 1972; Gunning, 1977). The cell wall invaginations that are distinctive of transfer cells dramatically increase the surface area to volume ratio of the plasma membrane, that, coupled with the mitochondrial abundance, serve to enhance these cells’ nutrient transport capacity (**Figures 3D,E**). As such, transfer cells have been functionally linked to nutrient acquisition (Gunning and Pate, 1969), secretion (Faraday and Thomson, 1986), loading and unloading from the vascular network for nutrient allocation and partitioning (Watson et al., 1977; Ligrone and Gambardella, 1988; Wimmers and Turgeon, 1991; Turgeon et al., 1993; Haritatos et al., 2000; Knop et al., 2001), development of reproductive tissues (Pate and Gunning, 1972), and delivery of nutrients between generations (Ligrone and Gambardella, 1988; Ligrone et al., 1993). Given the nutrient dependency of the sporophyte sink on the gametophyte source (**Figure 4B**), the presence of transfer cells in the haustorium (**Figures 3D,E**), and the localization of P-type ATPases in the transfer cells (**Figures 3F,G**), we postulated that the transfer cells at the sporophyte-gametophyte junction in *Physcomitrella* actively transport Suc across the filial boundary.

The few physiological studies on moss nutrient transport, however, have yielded contradictory results. While the whole set of Suc-metabolizing enzymes using the SUS pathway have been found to be enzymatically active in the sporophytes of mosses, Renault et al. (1989, 1992) instead found that the Suc gets irreversibly hydrolyzed into glucose and fructose by INVs at the highly acidic sporophyte-gametophyte apoplast (Renault et al., 1989, 1992). This would be plausible under the scenario that an irreversible hydrolysis of Suc into hexoses would create a Suc concentration gradient across the apoplasm, but counterintuitively, these hexoses then get absorbed into the haustorium without the use of proton motive force, and get reconverted into Suc prior to being assimilated (Renault et al., 1992). The proton motive force was found to be empowering the active uptake of amino acids instead. While the acidic pH of the apoplast is amenable to cell wall INV activity, one of the key pieces of evidence supporting Renault et al.’s proposition was the use of *p*-Chloromercuribenzenesulfonic acid (PCMBS), a non-permeant thiol reagent that modifies sulfhydryl groups, as an INV inhibitor (Renault et al., 1992). INV contains sulfhydryl groups in its active site (Krishnan et al., 1985), and as such PCMBS can act as an INV inhibitor, but this does not preclude the fact that PCMBS is also a potent inhibitor of apoplasmic loading (Giaquinta, 1979, 1983). As stated previously, the INV-based method of Suc uptake/re-synthesis into the sporophyte would be twice as energetically expensive as a SUS-based one. Assuming that mosses are not wasteful in their energy allocation, and predicated on our own evidence, we hypothesized that the P-type ATPases in the transfer cells (**Figures 3F,G**) generate a proton gradient across the apoplasm that drives the secondary active transport of Suc through SUTs into the transfer cells, wherein part of the Suc is catabolized via the SUS-pathway to, in turn, deliver the ATP required to maintain the proton gradient – analogous to what has been reported in apoplasmically phloem loading higher plants like *A. thaliana* (Haritatos et al., 2000; Pizzio et al., 2015).

In light of this, we tested whether 2,4-DNP would inhibit the transport of exogenously supplied [U^14^C]-sucrose into the heterotrophic sporangia of *Physcomitrella*, and found that this protonophore effectively diminished the [U^14^C]-sucrose-associated label taken up in the sporangia (**Figure 4C**). Congruently, application of a SUT-specific inhibitor DEPC (Supplementary Figure S3) (Bush, 1993) also reduced the uptake of radiolabel into the sporangia (**Figure 4C**). To further test our postulate that sucrose was being transported via SUTs, we applied exogenous [U^14^C]-maltose, and found that the associated radiolabel was also incorporated in the sporangia (**Figure 4C**). Phylogenetic evidence shows the existence of both types II and III SUTs in the *Physcomitrella* genome (Kühn and Grof, 2010; Reinders et al., 2012a), and experimental evidence shows that the type II SUTs have an affinity for maltose as well (Sivitz et al., 2005; Reinders et al., 2006, 2012b; Sun et al., 2009). Hence the preceding results, taken together, were consistent with an apoplasmic Suc loading mechanism into the *Physcomitrella* transfer cells.

The direct corollary of our aforementioned proposition is also the localization of key enzymes like SUS and H^+^PPase associated with the SUS-pathway (Pizzio et al., 2015; Regmi et al., 2015) in the transfer cells. Orthologs for both SUS (Supplementary Figure S4) (Regmi et al., 2015) and H^+^PPases (Supplementary Figures S3, S5)^9^ are found in the *Physcomitrella* genome, with five paralogs identified for the former (Supplementary Figure S4), and four for the latter (Supplementary Figures S3, S5). Using antisera generated against a highly conserved motif in H^+^-PPases (**Figure 5D** and Supplementary Figure S3) and cross-reactive antisera against SUS (Regmi et al., 2015), we then proceeded to immunolocalize H^+^-PPases and SUS in *Physcomitrella* gametophytes and sporophytes at both the light and electron microscopy level. Immunohistochemical localization of H^+^-PPases and SUS in the sporophytes revealed that both the enzymes were prominently expressed at the transfer cells lining the gametophyte-sporophyte junction (**Figures 5E–G,O**), supporting our argument that a SUS-pathway mediated Suc transport occurs at the haustorial junction. On the contrary, relatively weak signals were detected in the gametophore stem and leaves when probed for SUS (**Figure 5N**) and H^+^-PPases (**Figures 5A,B**), in agreement with a symplasmic mode of Suc transport in these gametophytic tissues. At the electron microscopic level, it was found that SUS was localized in proximity to the cell wall invaginations (**Figures 5S,T**), while H^+^-PPases were intriguingly found to be localized at both the plasma membrane and the vacuoles of the transfer cells (**Figures 5L,M**). SUS localization in the sieve element-companion cell (SE-CC) complex in rice minor veins (Regmi et al., 2015), and plasma membrane localization of H^+^-PPases in the SE-CC complexes of both rice and *Arabidopsis* (Paez-Valencia et al., 2011; Regmi et al., 2015) have been shown to be implicated in the SUS-pathway mediated active phloem loading scheme, and as such, further buttress our argument that an apoplasmic loading mechanism exists in a basal land plant lineage represented by *Physcomitrella*.

Though our evidence is consistent with a SUS-based ATP-conserving pathway as the most likely mechanism of Suc transport from the gametophyte to the sporophyte, we must also concede that this phenomenon might be stage-specific. In other words, the sporophytes analyzed in our experiment were immature but fully formed, and it cannot be ignored that there might be developmental stages wherein Suc metabolism might involve an acidic INV-based mechanism. For instance, it is known that a high ratio of hexoses to Suc promotes mitosis (Weber et al., 1995, 1996), and as such, at early stages of sporophyte differentiation and development, an INV mediated irreversible catalysis of Suc into glucose and fructose might occur. However, as previously mentioned, the INV orthologs in *Physcomitrella* cluster closer to their vacuolar counterparts rather than the acidic INVs in *Arabidopsis* (Thelander et al., 2009). Furthermore, a genome-wide transcriptomic analysis of the *Physcomitrella* sporophyte showed up-regulation of genes related to carbohydrate metabolism relative to the gametophyte (O’Donoghue et al., 2013). Specifically, the data revealed up-regulation of gene ontology identities directly related to sucrose metabolism when comparing gametophyte to mid-sporophyte or early sporophyte to mid-sporophyte stages, including – SUS activity (GO: 0016157); sucrose transport (GO: 0015770); sucrose metabolic process (GO: 0005985); sucrose transporter transmembrane activity (GO: 0008515); and energy-coupled proton transport (GO:0015985) (O’Donoghue et al., 2013). Therefore, the weight of evidence favors the model where sucrose is apoplasmically loaded into the *Physcomitrella* transfer cells.

**FIGURE 6:**
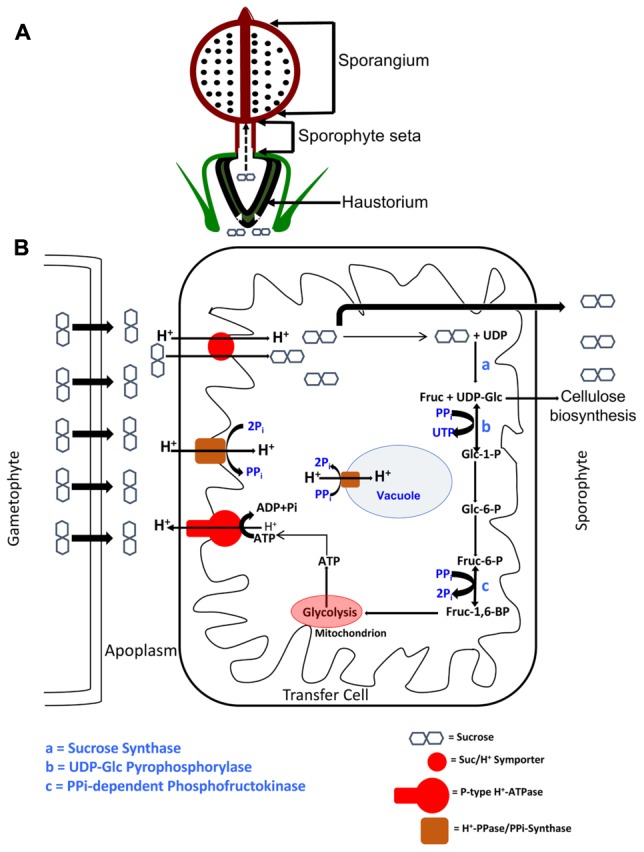
Schematic depiction of an ATP-conserving Sucrose Synthase-based apoplasmic sucrose loading scheme in the transfer cells of *P. patens*. **(A)** The sucrose photosynthesized by the gametophyte is symplasmically transported acropetally toward the heterotrophic sporophyte, where the haustorium, composed of transfer cells, presents a diffusion barrier. **(B)** The symplasmically transported sucrose is effluxed into the apoplasm via SWEET proteins, where an acidic pH is maintained by the action of P-type ATPases. This proton motive force is used by sucrose/H^+^ symporters (SUTs) to import sucrose into the transfer cells. Part of the symported sucrose enters the pyrophosphate (PPi)-dependent Sucrose Synthase (SUS) pathway, while the rest is transported to the sporophyte sink via seta. Two of the steps in this SUS pathway, depicted as b and c, and catalyzed by UDP-glucose pyrophosphorylase and PPi-dependent phosphofructokinase, respectively, operate near equilibria and are PPi-dependent. In other words, a supply of PPi, by Le Chatlier’s principle, drives these reactions forward, and ultimately toward respiration in the abundant mitochondria. The dual membrane localization of Proton Pyrophosphatase (H^+^-PPases) suggests this protein’s dual, and mutually inclusive, roles in PPi homeostasis as PPi-synthase at the plasma membrane and as PPi-hydrolase at the tonoplast. The UDP-glucose generated by the catalytic activity of SUS (step a) can also be used for cellulose biosynthesis in the growing sporophyte.

The set of evidence described herein is consistent with *Physcomitrella* utilizing a symplasmic mode of photosynthate transport in the gametophytic generation and then switching to an apoplasmic mode to transport photosynthate into the sink tissues of the sporophyte. We can thus conceive of a model (**Figure 6**) where (i) the Suc photosynthesized by the photoautotrophic gametophores moves symplasmically via plasmodesmata in the haploid generation, (ii) upon sporophyte induction, the Suc is acropetally transported from the gametophore, (iii) where, at the gametophyte-sporophyte junction, the Suc moves into the apoplasm, (iv) before being symported into the transfer cells lining this interface by SUTs, (v) the energy for which is provided by the proton motive force established by P-type ATPases, (vi) that, in turn, are empowered by the catabolism of some of the symported Suc via the pyrophosphate-dependent SUS pathway, wherein tonoplast/plasma membrane localized H^+^PPases/PPi-synthases maintain PPi homeostasis, (vii) while the rest of the Suc is transported to the heterotrophic sporangium through the sporophyte seta.

On a wider purview, this manuscript sheds some light on the physiological mechanisms underlying Suc partitioning in mosses, and in doing so, unearths interesting evolutionary parallels. Firstly, minor vein vascular cells in higher plants like *Arabidopsis* and the non-vascular food-conducting cells of *Physcomitrella* harbor plasmodesmatal pores that form symplasmic conduits through which photosynthesized Suc is translocated. Remarkably, in evolutionarily divergent organisms the pathway for passive diffusion of Suc is via pores that are seemingly both structurally and functionally homologous. However, Suc movement can also occur via the cell wall space in an energy-dependent process. The companion cells in minor veins of apoplasmically phloem loading species harbor SUS, a fully operational PPi-dependent glycolytic sequence, and a high density of mitochondria – molecular and cytological features that make them well-suited for a Suc concentrating and loading step in the phloem tissue. Is this necessarily a unique feature of some advanced lineages of Angiosperms? Bryophytes and the ancestor of vascular plants diverged early in land plant evolution, and despite their divergent morphologies and life cycles, the evidence points to the conservation of molecular pathways implicated in the metabolism of Suc. The enzymatic and morphological toolkit of companion cells finds its primordial counterpart in the transfer cells of *Physcomitrella*, wherein the same PPi-dependent SUS pathway and its associated enzymes seem to be implicated in the transfer of Suc from the photosynthetic gametophyte to its nutritionally reliant sporophyte. *Physcomitrella patens*, as a model basal land plant system with a well-tested repertoire of genetic tools, is the ideal candidate to pursue further research in this area.

## Author Contributions

KR and RG designed the experiments. KR performed all the experiments with assistance from LL in radioisotope labeling and fluorescent tracing. KR wrote the manuscript with help from LL and RG, and all authors approved the final version of the manuscript.

## Conflict of Interest Statement

The authors declare that the research was conducted in the absence of any commercial or financial relationships that could be construed as a potential conflict of interest.

## Footnotes

1 www.leica-microsystems.com

2 www.biogenex.com

3 www.cellsignal.com

4 www.dako.com

5 www.bio-rad.com

6 www.arabidopsis.org

7 www.uniprot.org

8 www.gramene.org

9 www.cosmoss.org

## Abbreviations

AHA3: *Arabidopsis* H^+^ ATPase 3
CFDA: 5(6)-carboxyfluorescein diacetate
DEPC: diethyl pyrocarbonate
H^+^-PPase: proton pyrophosphatase
HPTS: 8-hydroxypyrene-1,3,6-trisulfonic acid
P-type ATPase: plasma membrane-type ATPase
Suc: sucrose
SUS: sucrose synthase
SUT: sucrose/H^+^ symporter
SWEET: sugars will eventually be transported
2,4-DNP: 2,4-dinitrophenol
