# Supplementary material for: Alternate Modes of Photosynthate Transport in the Alternating Generations of Physcomitrella patens
Source: Front Plant Sci. 2017 Nov 13;8:1956. doi: 10.3389/fpls.2017.01956 (PMC5693889; doi:10.3389/fpls.2017.01956)
Supplement: Supplementary file 1 [file Image_1.PDF]

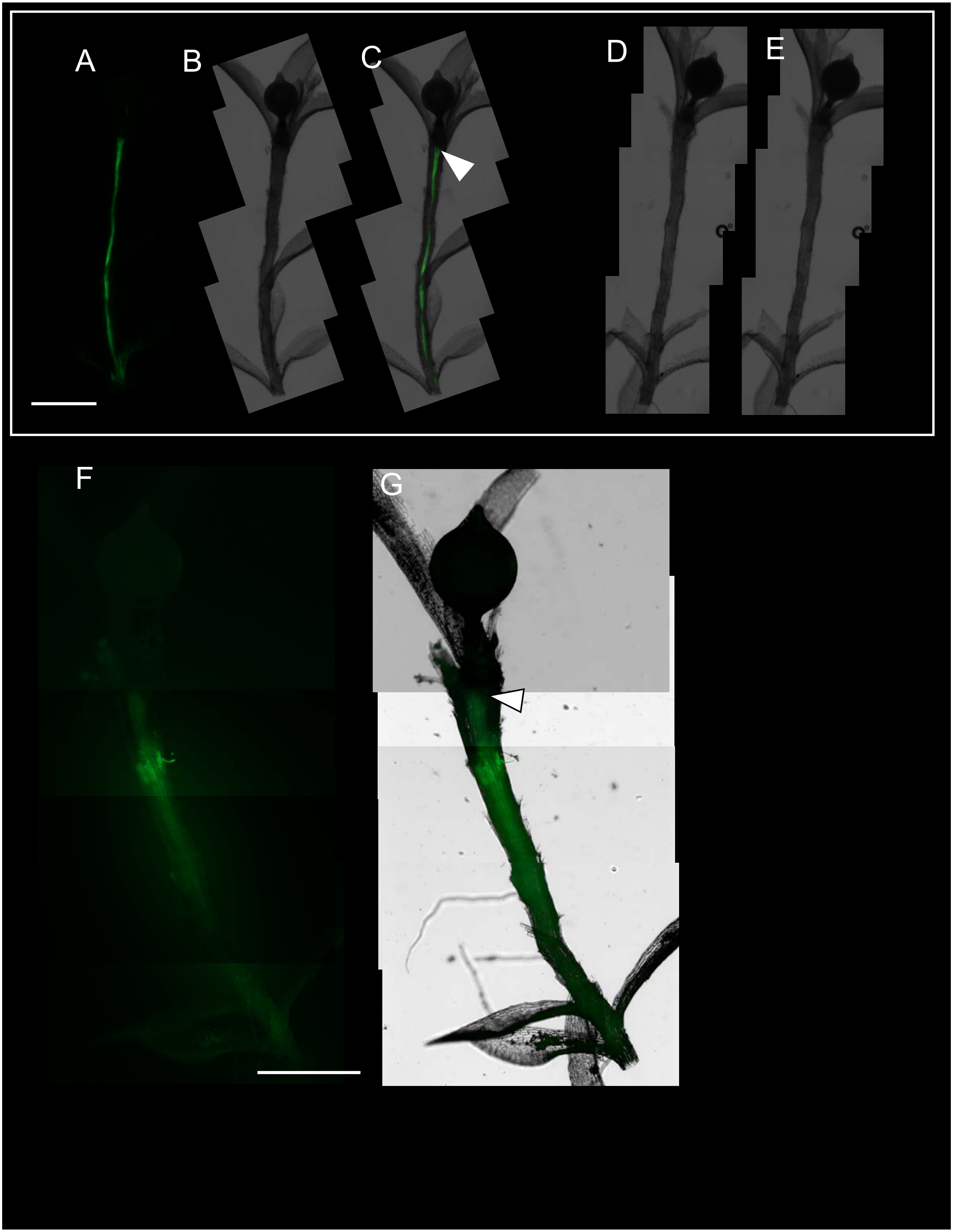
A
B
C
D
E
F
G

- **Figure S1: Symplasmic barrier separates the *Physcomitrella* sporophyte from the gametophyte.** (A) Montage of epifluorescent micrographs showing the movement of 8-Hydroxypyrene-1,3,6-trisulfonic acid (HPTS) to the gametophyte-sporophyte interface 3 hours after application from the rhizoidal end. (B) Brightfield micrographs of the sporangium-bearing gametophyte. (C) Overlay of (A) and (B) showing the interface where the HPTS movement stopped (arrowhead). (D & E) Representative negative control with assembled brightfield micrographs (D) and overlay (E) of the brightfield and GFP channels showing no interfering autofluorescence. (F & G) Epifluorescent micrographs showing the movement of 5(6)-carboxyfluorescein diacetate (CFDA) in a sporophyte-bearing gametophyte when 2,4-dinitrophenol (2,4-DNP) was also added. Following 1 hour pretreatment with 5mM 2,4-DNP, a mixture of 5mM 2,4-DNP and CFDA was applied from the rhizoidal end, and the moss imaged after 3 hours. Symplasmic movement of CFDA through the gametophyte stem was unimpeded, and was only stopped at the haustorial junction (arrowhead). Scale bars: (A – E) 2.5 mm, (F & G) 5 mm.

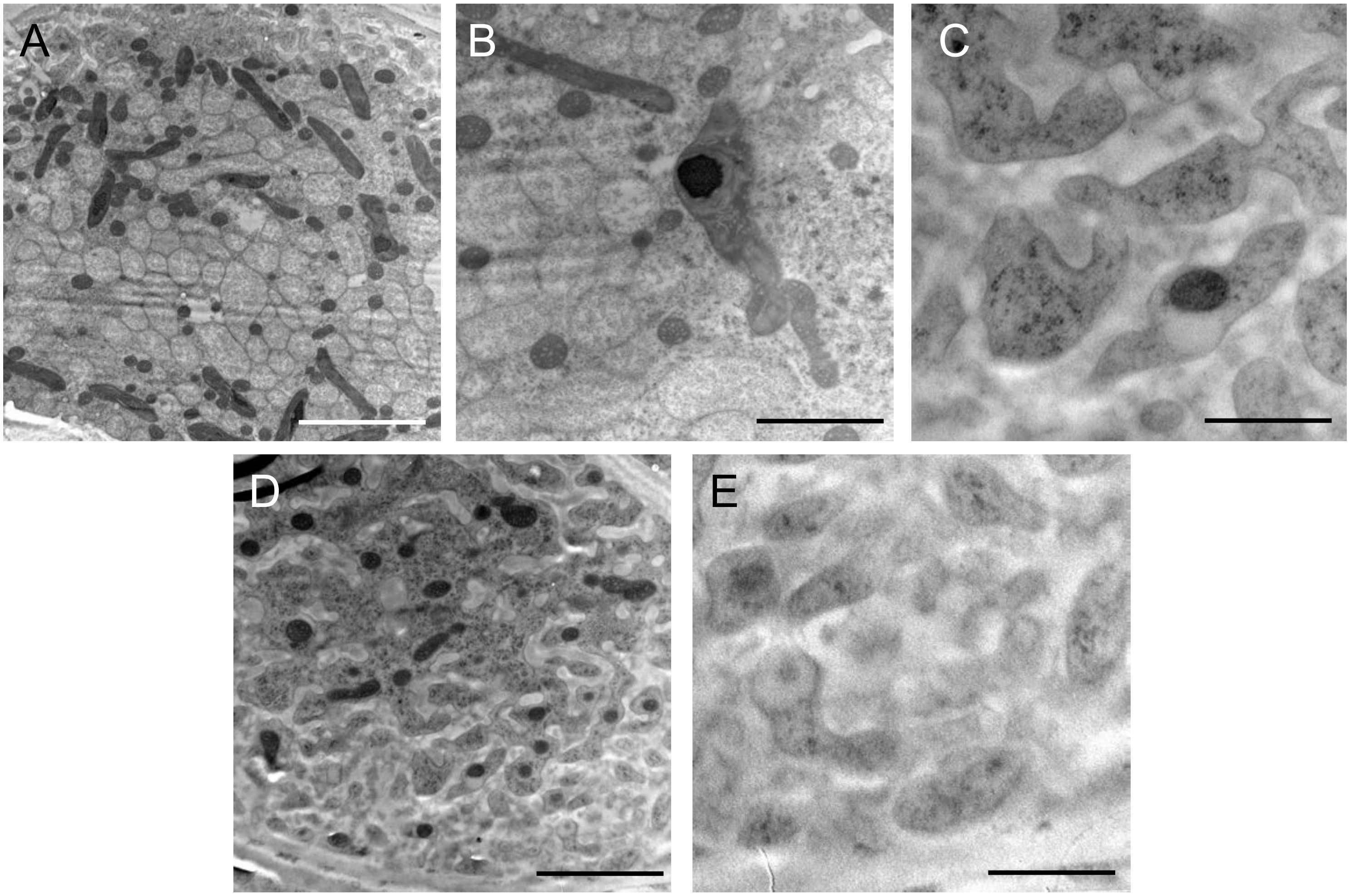

- **Figure S2: Representative negative controls for immunogold labeling of Proton Pyrophosphatases (H^+-PPases) and Sucrose Synthase (SUS) in high-pressure frozen/freeze substituted *Physcomitrella* transfer cells.** (A – C) Pre-immune serum controls for silver enhanced immunogold labeling for H^+-PPases showing no labeling at the vacuoles (B) or at the plasma membrane (C). (D – E) No immunogold labeling is observed at the plasma membrane when the primary antibody for SUS is excluded. Scale bars: (A) 5 µm, (B) 1 µm, (C) 250 nm, (D) 5 µm, (E) 300 nm.

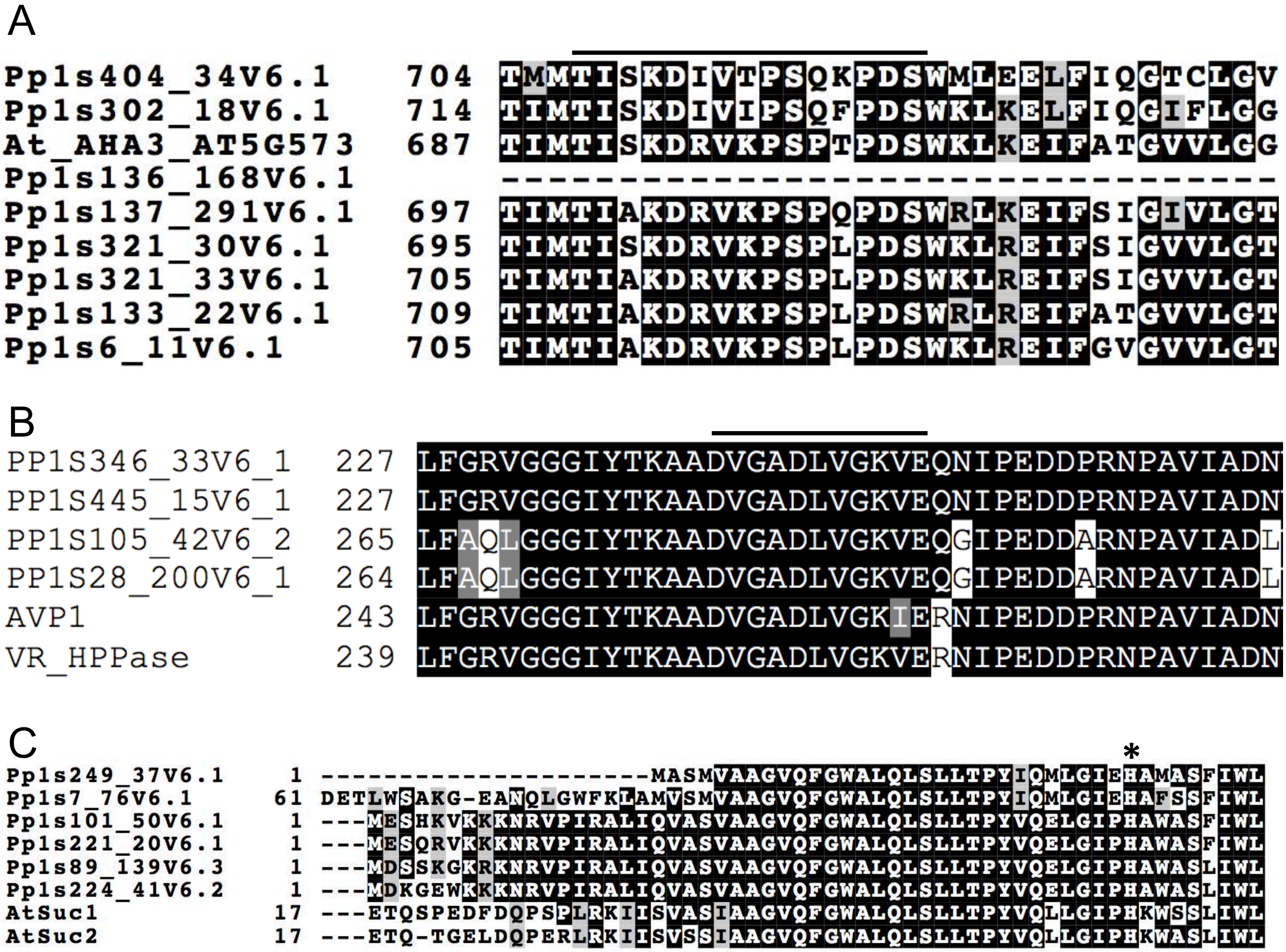

- **Figure S3: Clustal Omega amino acid alignments of orthologs of P-type ATPases, Proton Pyrophosphatases (H^+-PPases), and Sucrose Symporters (SUTs) in *Physcomitrella patens*.** (A) The *Arabidopsis thaliana* companion cell-specific AHA3 protein harbors a conserved epitope with the sequence TISKDRVKPSPTDSW (shown with a line) against which antibodies were generated in rabbit (Pizzio et al., 2015). This epitope is highly conserved in the AHA3 orthologs in *Physcomitrella* as well, confirming the protein bands recognized in the immunoblot (Fig. 3I). (B) H^+-PPases are highly evolutionarily conserved proteins. The pyrophosphate-binding domain with the conserved amino acid sequence $D(X)_7KXE$ (marked with a line) is highly conserved in species including *Vigna radiata* (VR_HPPase), *Arabidopsis thaliana* (AVP1), and *Physcomitrella patens*. The antibodies against H^+-PPases were generated using this very epitope. The recognition of H^+-PPase-specific bands in Fig. 5D western blot is hence confirmed. (C) Amino acid alignment of *Arabidopsis thaliana* SUTs (Suc1 and Suc2) with their orthologs in *Physcomitrella* shows that the Histidine residue (denoted with *) that is the site for diethylpyrocarbonate (DEPC) inhibition is conserved, adding confidence to the data shown in Fig. 4 C. The *Arabidopsis* protein sequences were obtained from www.arabidopsis.org, *Vigna radiata* sequence from www.uniprot.org, and *Physcomitrella* orthologs for the aforementioned proteins were determined by using the in-built BlastP function in either www.cosmoss.org, or www.gramene.org. The amino acid sequences were aligned using ClustalOmega (http://www.ebi.ac.uk/Tools/msa/clustalo/), and the figures assembled in Boxshade (http://www.ch.embnet.org/software/BOX_form.html).

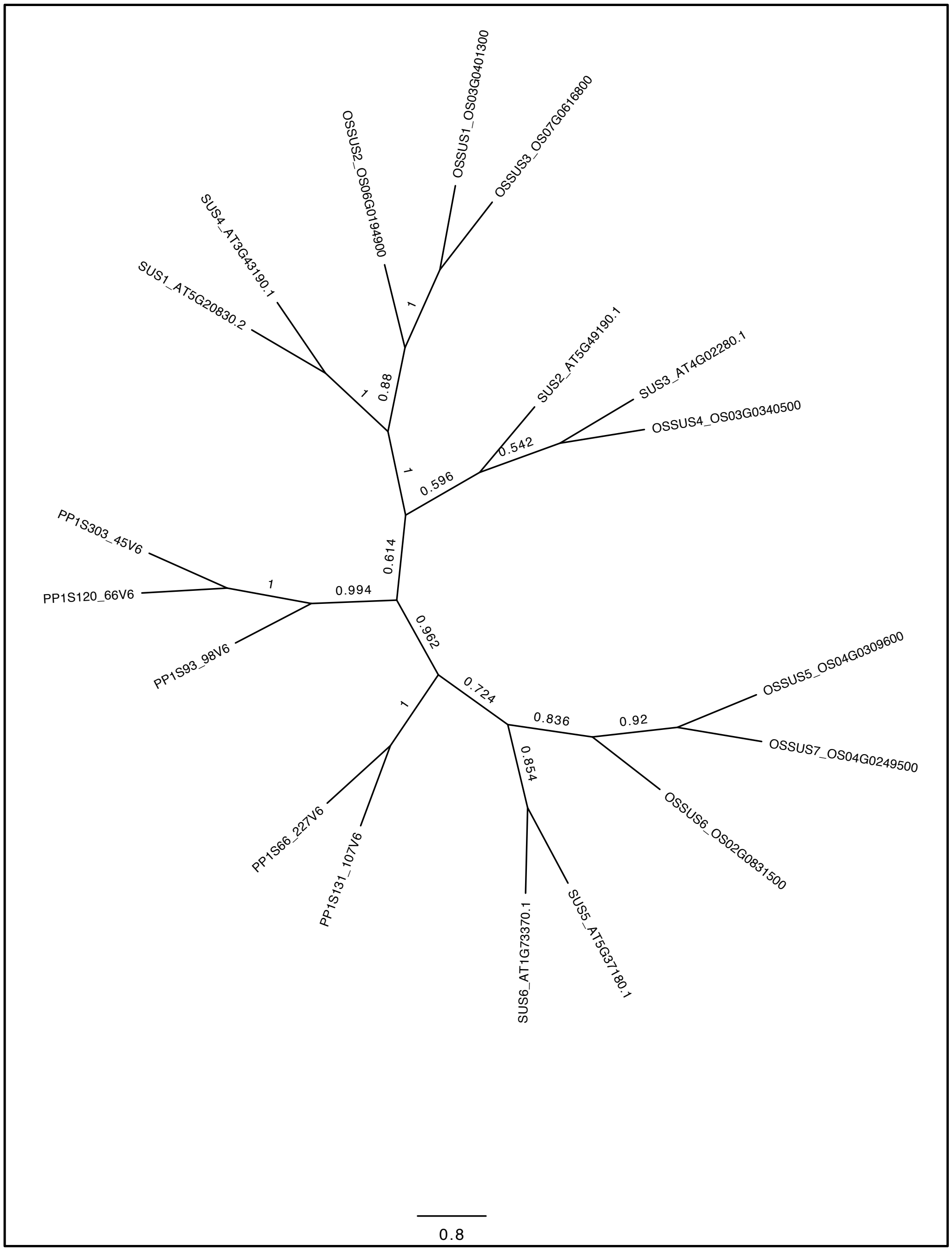

- **Figure S4: Molecular phylogenetic analysis of Sucrose Synthase (SUS) orthologs in *Physcomitrella* by Maximum Likelihood method conducted in MEGA5.** Amino acid sequence alignment of 5 putative SUS orthologs in *Physcomitrella patens* (PP) ßwas first performed against SUS sequences from *Arabidopsis thaliana* and *Oryza sativa* (rice) using MUSCLE. The evolutionary history of *Physcomitrella* SUS proteins was inferred by using the maximum likelihood method based on the JTT matrix-based model, and the bootstrap consensus tree inferred from 500 replicates is taken to represent the evolutionary history of the taxa analyzed. The percentages of replicate trees in which the associated taxa clustered together in the bootstrap test are shown next to the branches, with the inference that the SUS orthologs in *Physcomitrella* cluster in two distinct clades.

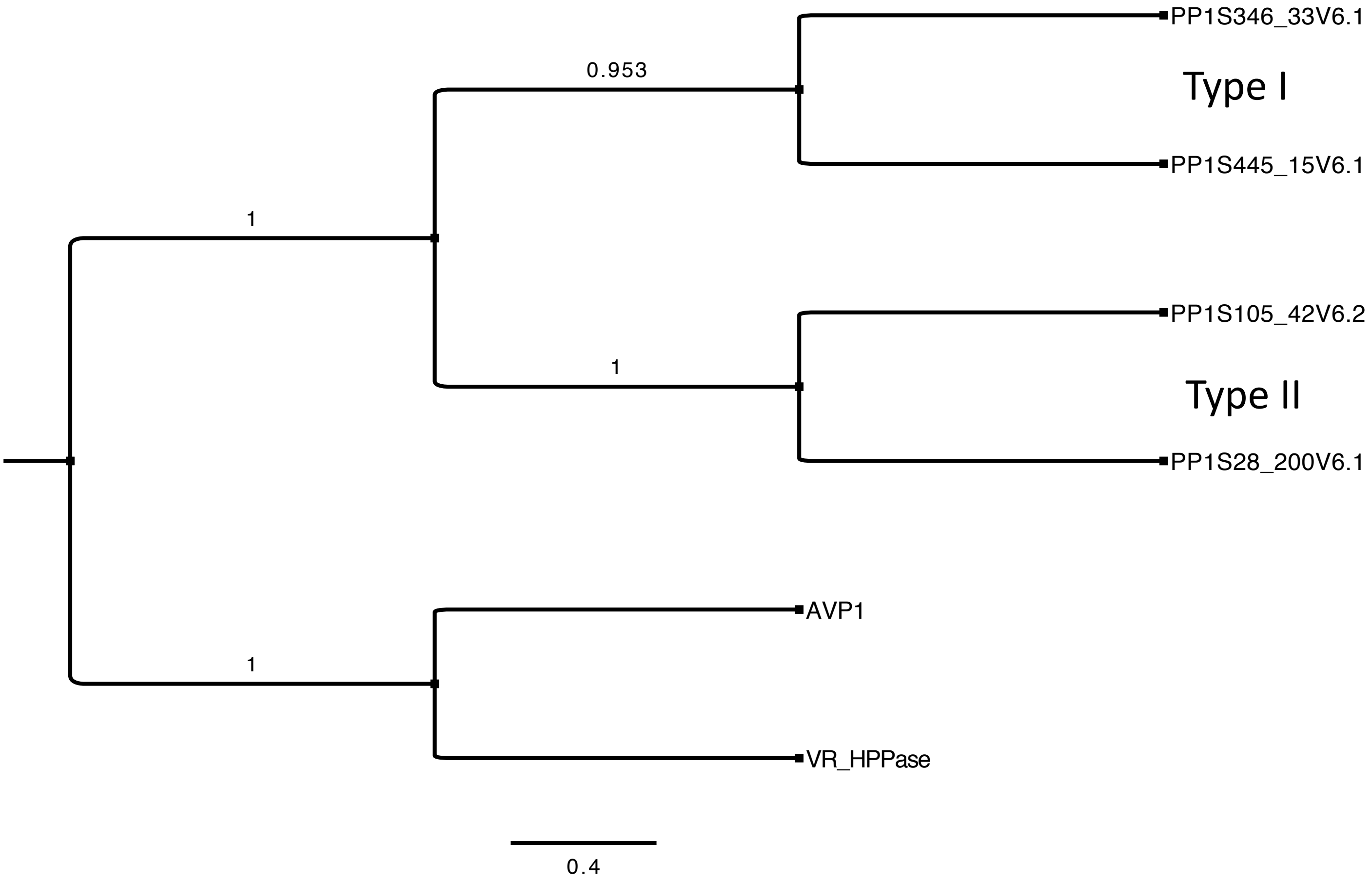

- **Figure S5: Molecular phylogenetic analysis of H^+-PPase orthologs in *Physcomitrella* by Maximum Likelihood method conducted in MEGA5.** Amino acid sequence alignment of putative H^+-PPase orthologs in *Physcomitrella patens* (PP), with H^+-PPase from *Arabidopsis thaliana* (AVP1) and *Vigna radiata* (VR_HPPase) using MUSCLE. The evolutionary history of *Physcomitrella* H^+-PPases was inferred by using the maximum likelihood method based on the JTT matrix-based model, and the bootstrap consensus tree inferred from 500 replicates is taken to represent the evolutionary history of the taxa analyzed. The percentages of replicate trees in which the associated taxa clustered together in the bootstrap test are shown next to the branches, with the inference that there are two distinct phylogenetic clades of H^+-PPases in *Physcomitrella* – type I and type II, like in its angiosperm counterparts *Arabidopsis thaliana*.
